# Glycemia reduction in type 2 diabetes—Hypoglycemia outcomes: A randomized clinical trial

**DOI:** 10.1371/journal.pone.0309907

**Published:** 2024-11-15

**Authors:** Elizabeth R. Seaquist, Lawrence S. Phillips, Alokananda Ghosh, Chelsea Baker, Richard M. Bergenstal, Jill P. Crandall, Robin S. Goland, Michaela R. Gramzinski, Sophia H. Hox, Daniel S. Hsia, Mary L. Johnson, John M. Lachin, Philip Raskin, Willy M. Valencia, Andrea H. Waltje, Naji Younes, for the GRADE Research Group

**Affiliations:** 1 Department of Medicine, Division of Diabetes and Endocrinology, University of Minnesota Medical School, Minneapolis, MN, United States of America; 2 Department of Medicine, Atlanta VA Medical Center, Decatur, GA and Division of Endocrinology and Metabolism, Emory University School of Medicine, Atlanta, GA, United States of America; 3 Department of Biostatistics and Bioinformatics, Milken Institute of Public Health, The Biostatistics Center, The George Washington University, Rockville, MD, United States of America; 4 Department of Medicine, Division of Endocrinology, Metabolism and Diabetes, University of Colorado School of Medicine, Aurora, CO, United States of America; 5 Health Partners Institute, International Diabetes Center, Minneapolis, MN, United States of America; 6 Division of Endocrinology and Fleischer Institute for Diabetes & Metabolism, Albert Einstein College of Medicine, Bronx, NY, United States of America; 7 Departments of Medicine and Pediatrics, Naomi Berrie Diabetes Center, Columbia University, New York, NY, United States of America; 8 Department of Veterans Affairs Pacific Islands Health Care System, Honolulu, HI, United States of America; 9 Clinical Trials Unit, Pennington Biomedical Research Center, Baton Rouge, LA, United States of America; 10 University of Texas—Southwestern Medical Center, Dallas, TX, United States of America; 11 Geriatric Research Education and Clinical Center, Bruce W. Carter Veterans Affairs Center, Miami, FL, United States of America; 12 Department of Public Health Sciences, University of Miami, Miami, FL, United States of America; 13 Robert Stempel College of Public Health, Florida International University, University Park, FL, United States of America; 14 Endocrinology & Metabolism Institute, Center for Geriatric Medicine, Cleveland Clinic, OH, United States of America; 15 University of Michigan, Ann Arbor, MI, United States of America; Keio University School of Medicine, JAPAN

## Abstract

**Objective:**

Hypoglycemia is a major concern in type 2 diabetes (T2DM), but little is known about its likelihood compared across common therapies. We compared the likelihood of hypoglycemia among metformin-treated patients with T2DM randomized to the addition of one of 4 common therapies.

**Research design & methods:**

Randomized, controlled trial of 5,047 participants with T2DM of <10 years’ duration, hemoglobin A1c (HbA1c) 6.8–8.5% (50.8–69.4 mmol/mol). Randomization to addition of glargine U100, glimepiride, liraglutide, or sitagliptin over 5.0 ± 1.3 (mean ± SD) years. HbA1c was measured quarterly; if a level >7.5% (>58.5 mmol/mol) was confirmed, rescue glargine and/or aspart insulin was added. We conducted a per-protocol analysis of 4,830, who attended at least one post-baseline visit and took at least one dose of assigned study medication. We assessed severe hypoglycemia events reported throughout the entire study. At quarterly visits, all participants were asked about hypoglycemic symptoms within the last 30 days, and those in the glargine and glimepiride groups were asked for any measured glucose <70 mg/dL (3.9 mmol/L) within this time period.

**Results:**

While participants were taking their assigned medications, severe hypoglycemia occurred in 10 (0.8%), 16 (1.3%), 6 (0.5%), and 4 (0.3%), (p<0.05) and hypoglycemic symptoms in 659 (54.2%), 833 (68.3%), 375 (32.4%), and 361 (29.1%) of participants following randomization to glargine, glimepiride, liraglutide, and sitagliptin, respectively (p<0.001).

**Conclusions:**

In metformin-treated patients with T2DM who add a second medication, hypoglycemia is most likely with addition of glimepiride, less with glargine, and least likely with liraglutide and sitagliptin.

**Trial registration:**

ClinicalTrials.gov Identifier: NCT01794143.

## Introduction

Hypoglycemia can be the limiting factor in achieving acceptable target glycemic control in patients with diabetes. Hypoglycemia can be serious–more common than hyperglycemia as a cause of hospital admissions among Medicare beneficiaries in 1999–2011 [1], and hypoglycemia related to use of insulin or sulfonylureas was among the most frequent causes of drug-related adverse events in emergency departments in 2013–2014 [2]. Although less common, hypoglycemia has also been reported with standard dosages of metformin, liraglutide and sitagliptin monotherapy [3–5]. The relative risk of experiencing hypoglycemia with different classes of drugs used to treat type 2 diabetes has not been extensively studied. In the CAROLINA trial, subjects randomized to glipizide had significantly more hypoglycemia than did those randomized to linagliptin and in UKPDS those randomized to sulfonylurea [glipizide or chlorpropamide) plus ultralente insulin had less hypoglycemia than did those randomized to ultralente insulin alone [6,7]. Our understanding of the likelihood of hypoglycemia with commonly used drugs would be enhanced by high-quality, prospective studies.

The Glycemia Reduction Approaches in Diabetes: A Comparative Effectiveness Study (GRADE) permits such a direct comparison. In GRADE, hypoglycemia was assessed in participants with type 2 diabetes treated only with metformin and then randomized to the addition of insulin glargine U100, glimepiride, liraglutide, or sitagliptin [8,9]. Participants were queried about episodes of hypoglycemia and related symptoms at regular intervals. As previously reported, severe hypoglycemia was more common in those randomized to glargine or glimepiride compared to liraglutide or sitagliptin [8]. In this analysis, we expand our observations to examine the comparative likelihood of different categories of hypoglycemic events across treatment groups in a randomized, controlled trial where medications were used as in usual clinical practice.

## Research design and methods

As described previously [8,9], GRADE examined the addition of a long-acting insulin (glargine U100), a long-acting sulfonylurea (glimepiride), a glucagon-like peptide-1 receptor agonist (GLP-1 RA, liraglutide), or a dipeptidyl peptidase-4 inhibitor (DPP4, sitagliptin), in people with type 2 diabetes who were taking only metformin at baseline. Eligibility included duration of diabetes <10 years, age >30 years at diagnosis (>20 years in Native Americans), and baseline hemoglobin A1c (HbA1c) 6.8–8.5% (50.8–69.4 mmol/mol). Additional details on randomization and masking are provided in S2 Text. Institutional Review Board approval was obtained at each participating institution, and all participants gave written informed consent. Recruitment began May 1, 2013, and ended August 31, 2017.

Study medications were randomly assigned, and glargine and glimepiride were titrated with protocol-defined algorithms based on self-monitored blood glucose (SMBG) levels, aimed to achieve pre-breakfast glucose levels of 80–130 mg/dL (4.4–7.2 mmol/L) without hypoglycemic symptoms, or up to the maximum approved dose, whichever dose was lower. Liraglutide was titrated to 1.8 mg/day, unless limited by tolerability, and the sitagliptin dose was based on renal function according to the package insert. HbA1c was measured quarterly and if a value of > 7.5% (>58.5 mmol/mol) was confirmed, those randomized to glimepiride, liraglutide, or sitagliptin added “rescue” glargine, while those randomized to glargine added pre-prandial insulin aspart. Participants in the glargine and glimepiride groups were expected to perform SMBG with study-supplied strips and meters. During periods of glargine dose titration, participants were asked to check blood glucose in the morning when fasting. During periods of glimepiride dose titration, participants were asked to check blood glucose one to two times per day. Once doses of glargine and glimepiride were stabilized, both groups were asked to check blood glucose at least twice a week. Participants in these groups were also asked to perform SMBG if they had symptoms of hypoglycemia. At the discretion of study staff, SMBG could be performed more frequently and also used in the liraglutide and sitagliptin groups. SMBG was also implemented in participants who were given insulin after failing to maintain HbA1c ≤ 7.5% (≤ 58.5 mmol/mol).

**Fig 1:**
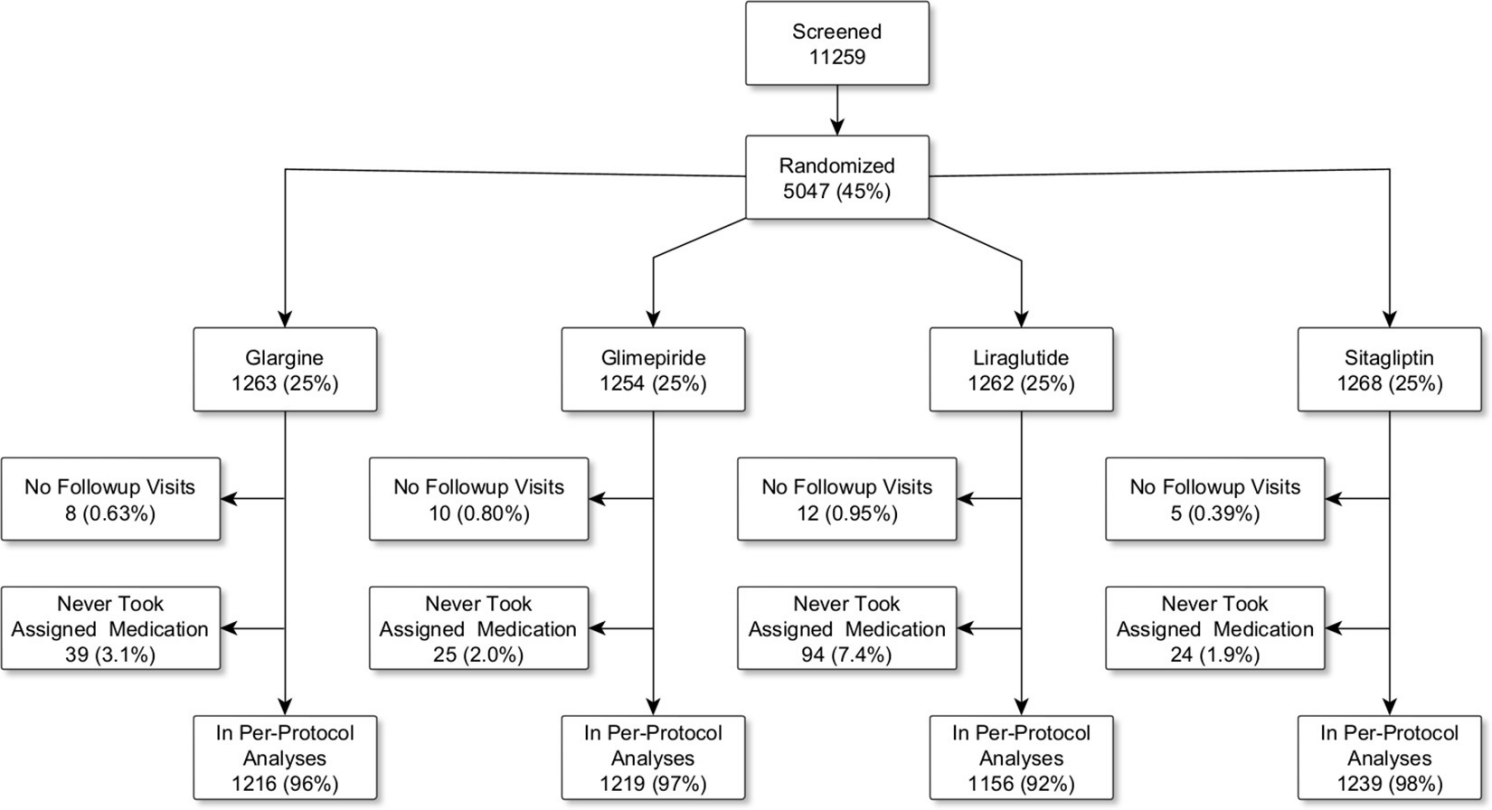
Consort diagram.

We enrolled 5,047 participants and report findings from a per-protocol subset of 4,830 (95.7%) participants, which excludes 217 participants who never took their assigned medication or never attended follow-up visits. The median observation period was 3.8 (range 0–7.4) years, 51% of the participants had at least 4 years and 68% had at least 3 years of follow-up (see CONSORT diagram, Fig 1).

### Hypoglycemia definition

The primary hypoglycemia outcome in GRADE was adjudicated severe hypoglycemia. All reports of severe hypoglycemia were collected throughout the study. In addition, at quarterly visits, all participants were asked about hypoglycemic symptoms within the last 30 days, and those in the glargine and glimepiride groups were asked for any measured glucose ≤70 mg/dL (3.9 mmol/L) within this time period. All reports of severe hypoglycemia triggered an adjudication process by investigators masked to treatment assignment (S3 Text). Only those episodes that were adjudicated as severe are included in this report.

### Statistical analysis

We conducted a "per-protocol" analysis of episodes of hypoglycemia occurring while participants were taking only metformin and their assigned study medication. This analysis was limited to the subset of participants who attended at least one post-baseline visit and took at least one dose of their assigned study medication (N = 4,830), and we censored any episode of hypoglycemia that occurred after the addition of rescue insulin, after any use of non-study diabetes medication, or the discontinuation of the assigned treatment. For categorical variables, differences in the counts and percentages by treatment group were evaluated with a chi-squared p-value comparing the groups. For continuous variables, differences in the mean ± standard deviation were evaluated with an F-test p-value comparing the groups.

The probability that a participant reported hypoglycemia at a quarterly visit was estimated using a logistic Generalized Estimating Equations (GEE) model with treatment group as the sole covariate, and a first-order autoregressive (AR(1)) correlation structure. The estimated probabilities within each assigned medication treatment group (expressed as a percentage) are reported along with their 95% asymptotic confidence interval. Kaplan-Meier (KM) cumulative incidence plots with log-rank tests were used to compare the four treatment arms for time to first severe hypoglycemia.

To assess the likelihood of hypoglycemia within each of seven pre-specified subgroups (age, sex, race, ethnicity, HbA1c, BMI, and diabetes duration), we again calculated the probability of a participant reporting hypoglycemic symptoms at a quarterly visit using logistic GEE models, with each medication treatment group, a single subgroup variable and a subgroup by treatment interaction. The estimated probability (expressed as a percentage) within treatment group and subgroup strata and its 95% asymptotic confidence interval are reported. The p-value is that of the treatment by subgroup interaction term in the GEE model using a multivariate Wald test.

All analyses were conducted with R 4.2.1 (R Core Team 2022). All tests were two-sided, with statistical significance set at p<0.05. All p-values were nominal with no adjustment for multiple comparisons.

### Ethics

Institutional Review Board approval was obtained at each participating institution, and all participants gave written informed consent. GRADE is a multi-center RCT, approved by each clinical site’s institutional review boards. Please refer to S1 Table for information on each clinical site’s review board. We have indicated the primary review board submission information from the most recent annual renewal: The George Washington University. Office of Human Research—Institutional Review Board; IRB Number: 071245; Last Approved: 7/25/2023; Expires: 8/23/2024.

## Results

The 4,830 per-protocol participants randomized to the glargine, glimepiride, liraglutide and sitagliptin treatment groups had similar characteristics at baseline [8,9]. The cohort was 36.1% female, 66.3% White, 19.4% Black, and 18.3% Hispanic with an average age of 57.1 years, body mass index (BMI) 34.3 kg/m^2^, duration of diabetes 4.2 years, and HbA1c 7.5% (58.4 mmol/mol) (Table 1).

**Table 1:** Baseline characteristics of the 4830 GRADE participants included in the per-protocol analyses.

*Demographics*						
N	4,830	1,216	1,219	1,156	1,239	p-value
Age at baseline visit (years)	57.1 *±* 10.0	57.0 *±* 9.9	57.0 *±* 10.0	57.3 *±* 9.9	57.3 *±* 10.1	0.855
Age group (years)						0.423
* <*45 years	593 (12.3%)	145 (11.9%)	159 (13.0%)	145 (12.5%)	144 (11.6%)	
* *45–59 years	2,231 (46.2%)	585 (48.1%)	541 (44.4%)	515 (44.6%)	590 (47.6%)	
* *60+ years	2,006 (41.5%)	486 (40.0%)	519 (42.6%)	496 (42.9%)	505 (40.8%)	
Female sex	1,743 (36.1%)	433 (35.6%)	463 (38.0%)	390 (33.7%)	457 (36.9%)	0.163
Race						0.360
* *All others	691 (14.3%)	167 (13.7%)	170 (13.9%)	171 (14.8%)	183 (14.8%)	
* *Black	939 (19.4%)	235 (19.3%)	262 (21.5%)	224 (19.4%)	218 (17.6%)	
* *White	3,200 (66.3%)	814 (66.9%)	787 (64.6%)	761 (65.8%)	838 (67.6%)	
Ethnicity						0.668
* *Non-Hispanic	3,919 (81.7%)	998 (82.7%)	978 (81.2%)	946 (82.1%)	997 (81.0%)	
* *Hispanic	876 (18.3%)	209 (17.3%)	227 (18.8%)	206 (17.9%)	234 (19.0%)	
Education completed						0.149
* *<High school	343 (7.1%)	88 (7.2%)	98 (8.0%)	72 (6.2%)	85 (6.9%)	
* *HS graduate	980 (20.3%)	231 (19.0%)	246 (20.2%)	242 (20.9%)	261 (21.1%)	
* *Some * *college	1,403 (29.1%)	385 (31.7%)	342 (28.1%)	349 (30.2%)	327 (26.4%)	
* *College degree or above	2,103 (43.5%)	512 (42.1%)	533 (43.7%)	493 (42.6%)	565 (45.6%)	
Blood pressure (BP)						
* *Systolic (mmHg)	128.3 ± 14.7	128.5 ± 14.9	128.2 ± 14.4	128.3 ± 14.9	128.3 ± 14.8	0.980
* *Diastolic (mmHg)	77.3 ± 9.8	77.5 ± 10.0	77.1 ± 9.5	77.5 ± 9.9	77.3 ± 9.8	0.699
* *History of hypertension	3,195 (66.1%)	807 (66.4%)	814 (66.8%)	772 (66.8%)	802 (64.7%)	0.668
Diabetes						
* *BMI (kg/m2)	34.3 ± 6.8	34.4 ± 6.8	34.3 ± 6.8	34.5 ± 6.7	34.1 ± 6.7	0.516
* *Duration of diabetes (years)	4.2 ± 2.7	4.2 ± 2.7	4.3 ± 2.8	4.2 ± 2.7	4.2 ± 2.7	0.504
* *HbA1c (%)	7.5 ± 0.5	7.5 ± 0.5	7.5 ± 0.5	7.5 ± 0.5	7.5 ± 0.5	0.692
* *HbA1c (mmol/mol)	58.4 ± 5.3	58.4 ± 5.2	58.2 ± 5.2	58.5 ± 5.4	58.5 ± 5.3	0.692
Renal						
* *eGFR (mL/min/1.73m2)	94.8 ± 16.8	94.6 ± 16.7	95.2 ± 16.9	94.3 ± 17.3	95.3 ± 16.3	0.360
* *eGFR < 60	120 (2.5%)	34 (2.8%)	27 (2.2%)	32 (2.8%)	27 (2.2%)	0.634
* *Urine ACR median/IQR (mg/g)	6.4 [3.0, 17.0]	6.7 [3.2, 18.0]	6.1 [2.9, 16.7]	6.6 [3.1, 18.7]	6.1 [3.1, 15.1]	0.525
* *Moderately elevated albuminuria	688 (14.3%)	170 (14.0%)	170 (14.0%)	176 (15.3%)	172 (13.9%)	0.749
* *Severely elevated albuminuria	83 (1.7%)	17 (1.4%)	27 (2.2%)	21 (1.8%)	18 (1.5%)	0.377

While participants were taking their assigned study medications, severe hypoglycemia occurred in 10 (0.8%), 16 (1.3%), 6 (0.5%), and 4 (0.3%) participants randomized to glargine, glimepiride, liraglutide, and sitagliptin, respectively (p<0.05). Hypoglycemic symptoms occurred in 659 (54.2%), 833 (68.3%), 375 (32.4%), and 361 (29.1%), p<0.001, of the glargine, glimepiride, liraglutide, and sitagliptin groups, respectively. In the glargine and glimepiride groups, any hypoglycemia (severe hypoglycemia, hypoglycemia symptoms or measured glucose < 70 mg/dL) occurred in 765 (62.9%) and 915 (75.1%), respectively, p<0.001. Among the four treatment groups, the probability of hypoglycemic symptoms being reported at a quarterly visit was higher with glimepiride than the other groups (18.8% vs. 10.6% with glargine, 5.1% with liraglutide, and 5.0% with sitagliptin [Table 2]). A similar pattern was seen for severe hypoglycemia, albeit with much lower probabilities.

**Table 2:** Participants who experienced different categories of hypoglycemia by treatment group, as determined by per-protocol analysis*.

	Glargine	Glimepiride	Liraglutide†	Sitagliptin†
	(N = 1,216)	(N = 1,219)	(N = 1,156)	(N = 1,239)
**Cases, N (%)**				
* *Severe hypoglycemia^§^‡	10 (0.8%)	16 (1.3%)	6 (0.5%)	4 (0.3%)
**Participants who experienced hypoglycemic events on at least one visit (not mutually exclusive)**				
* *Hypoglycemic symptoms‡	659 (54.2%)	833 (68.3%)	375 (32.4%)	361 (29.1%)
* *BG between 70 and 54‡	522 (42.9%)	687 (56.4%)		
* *BG less than 54‡	73 (6.0%)	106 (8.7%)		
* *Symptoms only‡	311 (25.6%)	311 (25.5%)		
* *Any hypoglycemia‡	765 (62.9%)	915 (75.1%)		
* *No hypoglycemia‡	451 (37.1%)	304 (24.9%)		
**Probability of at least one event being reported during the 30 days prior to a quarterly visit, % (95% CI)**				
Hypoglycemic symptoms	10.6 (9.7, 11.5)	18.8 (17.6, 20.1)	5.1 (4.5, 5.8)	5.0 (4.4, 5.6)

Hypoglycemia events by subtype (including no hypoglycemia) are summarized as counts and column percentages for all participants. Since participants can experience multiple episodes of hypoglycemia of varying severity and type during the study, the rows in this table are not mutually exclusive. Refer to S3 Text for hypoglycemia definitions and data collection details.

*Since participants can experience multiple episodes of hypoglycemia of varying severity and type during the study, the rows in this table are not mutually exclusive.

§Severe hypoglycemia was collected at any time during the study, and was not limited to the 30 days prior to a visit.

†Liraglutide and sitagliptin groups are not included in some categories due to differential blood glucose monitoring within the glargine U100 and glimepiride groups.

‡Nominal p<0.05.

Since participants could experience multiple episodes of hypoglycemia of varying severity and type during the study, the rows in Table 2 are not mutually exclusive.

**Fig 2:**
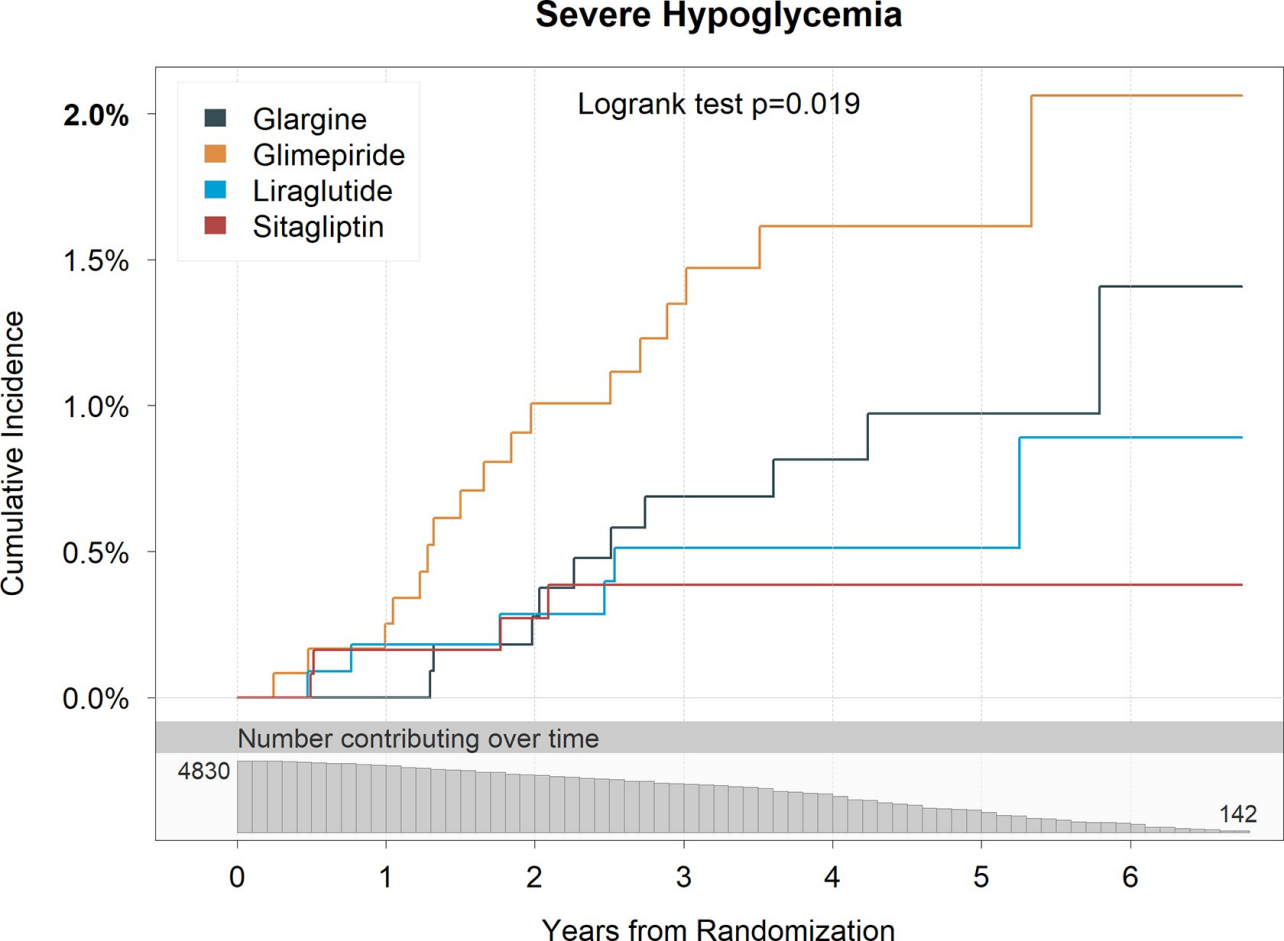
Cumulative incidence of severe hypoglycemia, by treatment group with attendant log rank test p-value of treatment group differences while on the assigned medication (per-protocol). The numbers plotted below the x-axis are the participants at risk for severe hypoglycemia at each follow-up time point by adjudication (i.e., the number of participants who were not adjudicated as having had severe hypoglycemia by that time).

Fig 2 shows the cumulative incidence of severe hypoglycemia. There was both more severe hypoglycemia and hypoglycemia occurring earlier in the study in those assigned to glimepiride compared to glargine, and less in those assigned to liraglutide and sitagliptin.

Of note, hypoglycemic symptoms were reported by participants in all groups (Table 2). Participants in the glargine and glimepiride groups measured their blood glucose in 77% of the episodes in which they had symptoms, and most of these symptomatic episodes were associated with blood glucose values less than 70 mg/dL. Close to one third of the participants in the liraglutide and sitagliptin groups (32.4% and 29.1%, respectively) reported hypoglycemic symptoms, but many did not have corresponding glucose values since they were not provided with testing supplies.

Subgroup analyses (Table 3) were conducted to assess potential impact on treatment group differences in the likelihood of reporting hypoglycemic symptoms at a quarterly visit, stratified by seven baseline factors. Probabilities (expressed as percentages) within treatment groups and among strata are presented. The pattern of treatment group differences varied across HbA1c tertiles’ strata (p = 0.04 for test of interaction). Among those in the lowest HbA1c tertile (6.8–7.2%), the likelihood was lower with glargine or glimepiride (8.7% for glargine, 18.6% for glimepiride) than for the other treatment groups (around 5.0%). None of the other factors (age, sex, etc.) showed heterogeneity among strata.

**Table 3:** Subgroup analyses of hypoglycemic symptoms stratified according to seven pre-specified baseline factors: age, sex, race, ethnicity, HbA1c, BMI, and diabetes duration. Here, N denotes the total number of participants in the stratum and treatment group; $N_{e}$ denotes the number of participants in the stratum and treatment group that experienced the event at least once, i.e. the number of cases in that stratum and treatment group; estimated probability (as a percentage) of hypoglycemic symptoms occurring in the 30 days prior to a quarterly visit with its asymptotic 95% confidence interval (CI); and the nominal p-value for the treatment by subgroup interaction term. HbA1c was the only subgroup with a statistically significant interaction (other subgroups are omitted).

Subgroup	Treatment	N	$N_{e}$	Probability* of at least one event being reported at a quarterly visit, % (95% CI)	Heterogeneity p-value
**HbA1c (%)**					0.040
* *6.8–7.2	Glargine	457	245	8.7 (7.5, 10.1)	
	Glimepiride	476	339	18.6 (16.7, 20.5)	
	Liraglutide	451	165	5.3 (4.4, 6.4)	
	Sitagliptin	455	146	4.9 (4.0, 6.1)	
* *7.3–7.7	Glargine	377	211	10.8 (9.3, 12.5)	
	Glimepiride	394	286	19.7 (17.7, 21.9)	
	Liraglutide	344	112	4.6 (3.7, 5.7)	
	Sitagliptin	401	130	4.7 (3.8, 5.8)	
* *7.8–8.5	Glargine	382	225	12.7 (11.0, 14.6)	
	Glimepiride	349	231	17.8 (15.6, 20.1)	
	Liraglutide	361	117	5.3 (4.2, 6.7)	
	Sitagliptin	383	110	5.4 (4.3, 6.7)	

*Probabilities are expressed as percentage, i.e. multiplied by 100.

## Conclusions

In this study of 4,830 participants with type 2 diabetes taking only metformin at baseline and randomized to addition of glargine, glimepiride, liraglutide, or sitagliptin, we found that while participants were on their assigned medication, there was significantly more severe hypoglycemia in those assigned to glimepiride than to glargine. Both medications had higher rates than with liraglutide or sitagliptin.

Severe hypoglycemia was rare, but hypoglycemic symptoms were common in the GRADE cohort. More than half of the participants assigned to addition of glargine and glimepiride and >25% of the participants in the liraglutide and sitagliptin groups reported these symptoms while they were taking only metformin and their assigned study medication. Since the symptoms participants report are not specific for hypoglycemia, a report of symptoms could lead to classification error if not confirmed with a glucose measurement. Only participants in the glargine and glimepiride groups were given glucose testing materials and provided with instructions for when to use them prior to meeting the HbA1c >7.5% (>58.5 mmol/mol) outcome. Thus, we cannot determine if the symptoms reported by participants randomized to liraglutide and sitagliptin truly reflect low glucose levels. More accurate ascertainment of hypoglycemia in future studies would ideally include more extensive confirmatory glucose testing.

Seven pre-specified baseline factors were examined to determine their potential contributions to the likelihood of reporting hypoglycemia symptoms at a quarterly visit, and only HbA1c was found to have a statistically significant interaction. This differs from results in the Action to Control Cardiovascular Risk in Diabetes (ACCORD) Study, where there was an increased risk of severe hypoglycemia in women and African Americans, and with advanced age, and higher baseline HbA1c [10]. However, ACCORD has not reported an association between baseline factors and the likelihood of hypoglycemic symptoms, and in GRADE the number of severe hypoglycemic events was too small to find a statistical relationship with baseline characteristics. Perhaps the reason we saw less hypoglycemia in those with the lowest baseline HbA1c levels is that they needed low dose glimepiride or glargine to achieve the treatment target.

Use of sulfonylureas and insulin carries a greater risk of hypoglycemia than diabetes medications acting via alternative mechanisms, including GLP-1 RAs such as liraglutide and DPP4 inhibitors such as sitagliptin [11,12]. However, most previous comparisons have not classified hypoglycemia across a spectrum from severe hypoglycemia to symptoms alone. Our finding that severe hypoglycemia and hypoglycemic symptoms were less common with liraglutide and sitagliptin than with glargine and glimepiride is consistent with previous reports [11,12] and clinical experience. This observation, when coupled with the finding that those randomized to liraglutide experienced less weight gain than those randomized to other medications and had an efficacy essentially equal to glargine and better than sitagliptin and glimepiride [8], provides further evidence that liraglutide might be considered as a first line agent to add on top of metformin in adults with type 2 diabetes of less than 10 years in duration. The presence of severe hypoglycemia in participants treated with liraglutide or sitagliptin was unexpected and provides evidence that this outcome can occur when these drugs are used in combination with metformin. However, there may have been other causes for hypoglycemia that were not captured by our adjudication process.

The strengths of this study include its large sample size; nationwide participation; and inclusion of individuals who may be at increased risk for iatrogenic hypoglycemia, including age 60+ years, lower level of education, and members of underrepresented racial/ethnic groups [10]. Further, treatment regimens were consistent with current clinical practice, and hypoglycemia was ascertained, categorized, and adjudicated using standardized procedures. The large sample size and successful randomization permitted a robust secondary analysis of an important clinical question.

Limitations include reliance on participant self-report for ascertainment of hypoglycemia, and potential undercounting of episodes since non-severe hypoglycemic events were only collected for the 30 days prior to the quarterly visits. However, recall bias was likely reduced by restricting the assessment to the 30-day window. Also, timing and cost precluded study of sodium-glucose cotransporter-2 (SGLT-2) inhibitors [9]. Therefore, GRADE cannot provide insight into the impact of this agent compared to the four study medications.

In conclusion, in adults with type 2 diabetes of less than 10 years duration using metformin monotherapy, the addition of glimepiride was associated with a greater risk of severe hypoglycemia by adjudication than addition of glargine, and both were associated with a greater risk than addition of liraglutide or sitagliptin. Parallel results were observed with less severe categories of hypoglycemia. If limiting the risk of hypoglycemia is a priority when optimizing glycemic management in patients with type 2 diabetes who are using only metformin, clinicians should consider adding a GLP-1 RA such as liraglutide, which has a low likelihood of hypoglycemia and high efficacy of glucose lowering [8]. If a sulfonylurea or insulin is needed, there may be less hypoglycemia with addition of glargine, compared to addition of glimepiride.

## Supporting information

S1 Checklist (PDF)

S1 Text GRADE research group listing. (PDF)

S2 Text GRADE masking and random assignment. (DOCX)

S3 Text Hypoglycemia data collection and definitions. A. Hypoglycemia data collection. B. Severe hypoglycemia adjudication. C. Hypoglycemia definitions.
(DOCX)

S1 Table IRB approval information. (DOCX)
