# Supplementary material for: Glycemia reduction in type 2 diabetes—Hypoglycemia outcomes: A randomized clinical trial
Source: PLoS One. 2024 Nov 15;19(11):e0309907. doi: 10.1371/journal.pone.0309907 (PMC11567630; doi:10.1371/journal.pone.0309907)
Supplement: S1 Text — (PDF) [file pone.0309907.s002.pdf]

CONFIDENTIAL

The **G**lycemia **R**eduction **A**pproaches in **D**iabetes: A Comparative **E**ffectiveness **Study (GRADE Study)**

VERSION 1.6.1

Date: March 17, 2017

GRADE Study Research Group

Sponsored by the National Institute of Diabetes and Digestive and Kidney Diseases (NIDDK)

GRADE Study Coordinating Center
Biostatistics Center
George Washington University
6110 Executive Boulevard
Rockville, Maryland 20852

PREFACE

The Glycemia Reduction Approaches in Diabetes: A Comparative Effectiveness Study (GRADE) Protocol describes the background, design, and organization of the study. The protocol will be maintained by the GRADE Coordinating Center over the course of the study through new releases of the protocol, or issuance of updates either in the form of revisions of complete chapters or pages, or in the form of supplemental protocol memoranda.

Summary of changes (other than correction of typographical errors, minor changes in wording, e.g. anti-diabetic medications changed to glucose-lowering medications, and changes in abbreviations) follows below:

Addendum, 08/24/2016.

Owing to a lag in the rate of recruitment, the period of recruitment has been extended to approximately 3.5 years and the total study duration extended to about 7.5 years, each by 0.5 years. The original protocol design would provide an average follow-up (time to end of study or loss to follow-up) of 4.8 years. With this extension, and allowing for an approximate 6 month delay in initiation of recruitment, the average follow-up is estimated to be slightly greater (4.9 years). Thus, throughout the text we have retained the 4.8 year follow-up since it is a lower bound on what might actually occur.

In addition, owing to the lag in recruitment, even with the approximate 6 month extension, we project that the final sample size could be as low as 4800. However, with the approximate 6 month extension of follow-up the excellent levels of power in the study will be preserved. Thus, we have not revised the protocol to present revised power computations for all the study outcomes. We do, however, state a sample size of 4800 to 5000 in places.

**Version 1.1**	**March 06, 2013**
**Location**	**Change**
Page 4, Section 4.0 (and throughout protocol)	The HbA1c range for inclusion has been changed from 6.5-8.5% to 6.8-8.5% as recommended by DSMB.
Page 8, Section 5.1	Sentence added to note that subjects who have been enrolled in phase 1 (screening and run-in) and have therefore been provided with study metformin, but who are ultimately not enrolled into the full scale trial, will be provided with metformin as they transition their diabetes care back to their own care provider.
Page 8, Section 5.1	Maximum run-in may be as long as 14 weeks

Page 13, Section 6.2	An additional exclusion has been added. It will exclude potential participants who have had a major CVD event or procedure during the previous year. This exclusion was recommended by the DSMB in order to decrease the theoretical risk of excess CVD mortality in GRADE that was seen in the ACCORD study. In ACCORD, participants with pre-existing CVD were at higher risk for excess CVD mortality when assigned to intensive therapy. We have pointed out in the protocol (page 9) and in the revised informed consent that GRADE differs from ACCORD in the study population (GRADE with substantially less diabetes duration and CVD risk factors and is expected to be younger), interventions (GRADE with fewer diabetes drugs than used in ACCORD in which >60% used $\geq$ 3 drugs) and glycemic goals (GRADE aiming for the currently recommended A1C <7% vs. ACCORD which aimed for <6%). Moreover, GRADE will exclude potential participants if they have had a CVD event or vascular procedure within previous year).
Page 2, Section 1.0 (and throughout protocol)	References to specific events/outcomes that will be "adjudicated" have been removed as being too specific for protocol. The adjudication process including the events that will be adjudicated will be included in the manual of operations.
Page 15, Table 1 (and throughout protocol)	Instead of a mixed meal tolerance test (MTT), a standard 75 gm oral glucose tolerance test (OGTT) will be performed.
Page 15, Table 1	A footnote indicates that measurements (blood pressure, height, weight, waist/hip circumference), peripheral neuropathy assessment, neurocognitive assessment, questionnaires may be administered at final run-in or baseline visit.
Page 18, Table 2	A footnote indicating that the DTSQ questionnaire will be administered at baseline, 6 months, and 1 year.<br>A footnote indicates that neuropathy assessment and questionnaires may be completed at final run-in or baseline visit.<br>Height measurement added at 4 years.<br>Health econ/utilities row deleted from table.
Page 31, Section 15.0	A phrase was added to clarify that any revisions to protocol would require an absolute two-thirds majority approval of Steering Committee members.
Page 16, Section 8.1<br>Page 25, Section 12.6	"Pancreatitis resulting in hospitalization" changed to "Pancreatitis" as all episodes of pancreatitis will be collected
Page 26, Section 13.2	Statistical method corrected from Hochberg to Holm
Page 30, Section 14.3	Two power calculations for subgroup analyses were corrected (89 to 94% and 59 to 69%), both showing more power.
Page 38, Reference 56	Statistical method reference changed.
**Version 1.2**	**August 30, 2013**
**Location**	**Change**

Page 4, Section 3.0	Two sentences have been added to the specific aims section for the comparison of cumulative incidence of cardiovascular disease, as well as incident cardiovascular events detected by ECG, among the study drugs
Page 9, Section 5.1	Revised language to clarify timing of the pre-randomization study visits, and minimum weeks between screening and baseline
Page 13, Section 6.2	3. Changed exclusion criteria to be more than 5 years of metformin treatment at time of screening (previously at time of randomization)
Page 13, Section 6.2	Added exclusion criteria about residing in the same household with another GRADE participant
Page 13, Section 6.2	History of cancer criterion moved from 15 to 14.
Page 13, Section 6.2	Changed the wording to clarify personal or family history of MEN-2 or medullary thyroid cancer
Page 15, Section 7.2	Revised language to clarify timing of the pre-randomization study visits, and targeted number of weeks between initial run-in and final run-in
Page 16, Table 1	Clarified that interim visits can be performed via phone or in-person visit, depending on titration schedule
Page 16, Table 1	Added electrocardiogram (ECG) measurement to the baseline visit procedures, with footnote that it may also be conducted at final run-in
Page 16, Table 1	Removed approximate week numbers that visits should occur, and reformatted to have visit type and visit activities in the table
Page 16, Table 1	Clarified that standard diabetes education can be performed at any run-in visit
Page 17, Section 8.1	Added new bullet points under "Cardiovascular" header to include ECG abnormality outcomes using the Minnesota ECG classification and to include assessment of cardiac autonomic function
Page 19, Table 2	Added new row for ECG measurement to be conducted at baseline visit, Year 2, Year 4, and Year 6 annual visits
Page 21, Section 10.1	Clarified reporting of SAEs for run-in and follow-up during the study.
Page 25, Section 12.5	Added paragraph under the section for Microvascular and Cardiovascular Outcomes for ECGs measurement of CV outcomes
Page 25, Section 12.6	Added several bullet points under the "Cardiovascular" sub-header to specify events that will be measured by ECG and to include incidence of cardiac autonomic dysfunction
Page 31, Section 15.0	Changed title of header to include policies
Page 31, Section 15.1	Added header for IRB Approval
Page 32, Section 15.2	Added header for Protocol Changes
**Version 1.3**	**January 01, 2014**
**Location**	**Change**
Page 1, Section 1.0	Changed the descriptions of the study population to reflect expanded eligibility criterion ("relatively" recently diagnosed type 2 diabetes, <10 years since diagnosis)

Page 2, Section 1.0	Modified the language used to describe the study population to reflect expanded eligibility criterion
Page 4, Section 3.0	Modified the language used to describe the study population to reflect expanded eligibility criterion
Page 5, Section 4.0	Changed the number of years since diagnosis in the description of the study population
Page 6, Figure 1	In the last line of the first box: Changed the number of years since diabetes diagnosis
Page 7, Figure 2	In the third oval: Replaced the word “endpoint” with “outcome”
Page 10, Section 5.2	Modified the language used to describe the study population to reflect expanded eligibility criterion
Page 10, Section 5.2	Changed “at bedtime” to “daily” in the description of initial dosage for patients assigned to basal insulin
Page 12, Section 6.0	Changed the descriptions of the study population to reflect expanded eligibility criterion (“relatively” recently diagnosed type 2 diabetes, <10 years since diagnosis)
Page 13, Section 6.1	Changed inclusion criterion to reflect increased number of years since diabetes diagnosis
Page 13, Section 6.2	Changed exclusion criterion to reflect increased number of years of metformin treatment
Page 13, Section 6.2	Clarified exclusion criterion to allow participants with a banding procedure reversed/removed after 1 year to be eligible.
Page 14, Section 6.2	Removed duplicate exclusion criterion: “History of cancer, other than non-melanoma skin cancer, that required therapy in the 5 years prior to randomization”
Page 14, Section 6.2	Added “acute hepatitis” to the revised exclusion criterion: “History of severe liver disease…”
Page 16, Table 1	Added language to include all blood glucose test sites in the description of Initial Run-in Visit activities
Page 22, Section 12.1	Clarified the definition of “maximally tolerated dose” to include participant preference
Page 22, Section 12.1	Added a paragraph describing the protocol for assessing the occurrence of the primary outcome in participants taking less than the maximally tolerated dose
Page 32, Section 15.1 (and throughout protocol)	Removed duplicate sentence noting the requirement of an absolute two-thirds majority approval by the Steering Committee for the passage of any protocol or consent amendments (duplicated in Section 15.2)
**Version 1.4**	**March 9, 2015**
**Location**	**Change**
Page 1, Section 1.0	Deleted “as well as at 3 years of follow-up” to allow approximate study durations. Added “and at intermediate time points”.
Page 5, Section 4.0	Corrected for accuracy that the secondary outcome is the time to the observation of an “HbA1c > 7.5%,” subsequently confirmed
Page 6, Section 4.0	Added language to indicate a planned follow-up of “approximately” 4 to 7 years

Page 7, Figure 2	Replaced with new Figure 2 to reflect the differences between the three groups randomized to the non-insulin drugs, and the glargine-assigned group related to secondary and tertiary outcomes
Page 7, Section 4.0	Added “Since the glargine-assigned group is already treated with glargine, they will essentially reach their secondary and tertiary outcome simultaneously.” As in new figure 2, clarifying the secondary and tertiary metabolic outcomes for the glargine assigned group.
Page 9, Section 5.1	Deleted “1000mg twice per day” to reflect that participants could be treated with metformin once per day
Page 10, Section 5.2	Added “or other dose-related side effects” to indicate that study doses may be adjusted for hypoglycemia or other side effects
Page 13, Section 6.2	Added “single use is allowed” to exclusion criteria #2 to reflect that a single dose of a hypoglycemia drug during the previous 6 months is not an exclusion
Page 14, Section 6.2	Replaced language in exclusion criteria #14 “history of cancer, other than non-melanoma skin cancer, that required therapy in the 5 years prior to randomization” to be clarified to “Any new diagnosis of cancer in the previous 5 years (other than non-melanoma skin cancer), or treatment for any cancer in the previous 5 years (other than non-melanoma skin cancer). Exceptions may be made, at the discretion of the local Principal Investigator and after review by the subcommittee overseeing protocol implementation, for cancers, such as some thyroid cancers, that have a benign clinical course and are not expected to interfere with conduct of the study.” This reflects a clarification of the eligibility criteria and the intent not to exclude participants with benign behaving malignancies that are very unlikely to limit lifespan, require therapy during the trial, or interfere with performance of the protocol.
Page 14, Section 6.2	Added language to exclusion criteria #20 “(and/or physiological replacement treatment are allowed, e.g. Addison’s disease)” to clarify this exclusion.
Page 14, Section 6.2	Added language to exclusion criteria #21 about treatment with atypical antipsychotics “known to be associated with a high risk of metabolic dysfunction” to clarify this exclusion since not all atypical antipsychotics are associated with metabolic disturbances.

Page 14, Section 6.2	Replaced language in exclusion criteria #25 “No non-study PCP or inability to identify such a PCP (who will provide non-study care) by the time of final run-in” to "At the time of final run-in, no identified PCP to provide non-study care. (Note: in cases where a study MD serves as the participant’s PCP, another study provider must assume GRADE management decisions for the participant during the study)” This change allows patients whose PCPs are part of the GRADE team to enroll in GRADE but separates the PCP care role from the GRADE care role, which is the intent of this eligibility criterion.
Page 15, Section 7.2	Added language “In some cases, such as temporary condition or blood test that resulted in an ineligible status, a repeat screening visit and/or blood test may be scheduled.”
Page 16, Table 1	Added language to the baseline randomization visit windows to indicate “approximately” 5-45 days from final run-in and 36-98 days from screening
Page 19, Table 2	A footnote indicating that the OGTT may be performed as often as annually. An OGTT may be performed at selected annual or quarterly visits, but no more than one OGTT will be performed per year.
Page 20, Section 8.2	Added language to indicate that the OGTT may be performed as often as annually. An OGTT may be performed at selected annual or quarterly visits, but no more than one OGTT will be performed per year.
Page 21, Section 12.1	Corrected language that if the initial HbA1c and confirmation value 3 to 6 weeks later are both >9%, the primary outcome “and secondary outcome” will have been reached.
Pages 21-22, Section 12.1	Replaced language to clarify that the primary outcome can only be reached after a minimum of 6 months of therapy, “unless the HbA1c at 3 months is >9% and is higher for the confirmation HbA1c 3-6 weeks later, in which case the primary and secondary outcomes will have been met at 3 months.” This represents a change in the outcome. The intent is to provide a window of time to allow the study medications to exert their effects and enough time for the HbA1c to equilibrate (the first 6-months) <u>unless</u> the HbA1c is >9% and rising. This approach is consistent with usual clinical care.
Page 22, Section 12.1	Deleted language “If, despite efforts on the part of the clinic team, the participant is below maximal tolerated dose at the time of primary outcome at 3 months, another 3 months will be allowed to achieve maximal dose. If after another 3-months, the participant is not at maximally tolerated dose for any reason, and has HbA1c $\geq$ 7%, this will count as the primary outcome. This will only apply to first 3 months of study.” to avoid confusion on study outcomes.
Pages 29-30, Section 14.0	Added language to indicate that the recruitment period is “approximately” 3 years, and that the follow-up period descriptions are also approximations

Page 31, Section 14.1	Added language to indicate that the recruitment period is "approximately" 3 years.
Page 31, Section 14.1	Corrected sample size from "4964" to "4968"
Page 35, Section 15.8	Added language to the study timeline description to indicate that the duration of the study, recruitment period, and follow-up period are approximate. Deleted specific language about "Screening will start in month 7 and the first patient will be randomized in month 10 of year 1" and "the last participant entered in month 9 of year 4" to allow for approximate study timelines
**Version 1.5**	**September 8, 2015**
**Location**	**Change**
Page 10, Section 5.2	Minor clarification to the starting dose of glargine (basal insulin) to state: "Glargine will be initiated with a dose of up to 20 units daily…" instead of "10 to 20 units daily…".
Page 12, Section 5.3	Minor clarification to the wording for accuracy to state: "At that time, intensive insulin therapy will be *initiated*" instead of "administered".
Page 13, Section 6.2	Revised language for "single use is allowed" to "limited use of no longer than seven days is allowed, for example during hospitalization".
Page 19, Table 2	Modified the table when the Oral Glucose Tolerance Test (OGTT) is performed from all annual visits to annual visits at 1, 3 and 5 years.<br>Revised footnote describing the OGTT frequency to indicate that an OGTT will be completed at baseline "and at the year 1, 3, and 5 annual visits. In addition, an OGTT will be performed at visits other than at 1, 3 and 5 years in subjects who have reached the confirmed primary outcome (cases) and in selected, matched subjects (controls) who will be tested at the same visit as the corresponding case." Previous wording stated that "OGTT may be performed annually at selected annual or quarterly visits, but no more than one OGTT will be performed per year."
Page 20, Section 8.2	Revised language about OGTT frequency to indicate that an OGTT will be completed at some annual visits to specify "and at the year 1, 3, and 5 annual visits." In addition, an OGTT may be completed at select annual or quarterly visits "other than at 1, 3 and 5 years in subjects who have reached the confirmed primary outcome (cases) and among selected, matched subjects (controls) who remain free of the primary outcome and who will be tested at the same visit as the corresponding case. Controls will be matched as closely as possible for general characteristics to a case including the assigned treatment, gender, baseline HbA1c, and time since randomization, among other factors."
Page 40, Bibliography and References	Replaced reference 60, with a newer statistical reference: "Lachin JM. Applications of the Wei-Lachin multivariate one-sided test for multiple outcomes on possibly different scales (2014) *PLoS ONE*, 9 (10), art. no. e108784."

Version 1.6	August 24, 2016
**Location**	**Change**
Preface	Added addendum to Project Summary for the extension in the recruitment and study duration (enrollment period to 3.5 years and duration of 7.5 years), and updated the size of the randomized cohort from 5000 participants to 4800-5000 (up to 5000) participants based on current recruitment trends.
Page 1, 5, 6, 13, 32, 33, 37	Updated the recruitment period to 3.5 years and study duration of 7.5 years. The size of the randomized cohort is updated from 5000 participants to 4800-5000 (up to 5000) participants.
Page 10, 11	Updated the lower range of fasting blood glucose for self-monitoring to 80 mg/dl from 70 mg/dl to be consistent with the 2016 American Diabetes Association guidelines. Current target range is 80 to 130 mg/dl. Updated reference for new guidelines.
Page 11	Addition of new section under Management of Glycemia to address management of participants whose HbA1c is greater than 8.5% for two measurements after being eligible to start insulin.
Page 13	Expanded enrollment at clinical centers from 150 participants to "about" 150 participants (up to 250) over 3.5 years.
Page 14	Updated exclusion criteria for renal function to be consistent with current treatment guidelines. Changed from serum creatinine levels to estimated GFR (eGFR) < 30 ml/min/1.73 m^2.
Page 16, 17, 22	Added eGFR next to serum creatinine for measure of renal function and noted that eGFR is calculated from serum creatinine.
Page 17	Added collection of stool sample in a subset of the cohort. Clarified references to the Central Biochemical Laboratory.
Page 18, 23, 27	Clarified definition of hypoglycemia and expanded definition of severe hypoglycemia to be consistent across sections of the protocol.
Page 20, 21	Addition of microbiome stool sample collection in a subset of GRADE participants at baseline and 6 months and potentially more often pending funding.
Page 21	Added column denoting the samples and assessments to be completed in Year 7 of the study. Removed footnote describing OGTTs case/control component of OGTT.
Page 22	Removed case/control component of OGTT and frequency of additional OGTTs beyond baseline, years 1, 3 and 5.
Page 32	Addition of addendum to Sample Size and Power section for the extension of recruitment from 3 years to 3.5 years and the follow-up from 7 years to approximately 7.5 years. Addition of statement that the study power is largely preserved with these changes.

Version 1.6.1	March 17, 2017
**Location**	**Change**
Page 8, Section 5	Added sentence regarding risk for vitamin B12 deficiency with metformin.
Page 11, Section 5.3	Added sentence noting that vitamin B12 levels will be tested once for all participants in 2017-18 and at year 4.
Page 13, Section 6.0	Have noted that the end of recruitment will occur in summer 2017 (previously was April 30, 2017).
Page 21, 22	Table 2 – added vitamin B12 measurements. Footnote added for testing at intervals depending on randomization date. Added footnote that ECGs will be conducted at selected annual visits during the study pending the availability of funding.
Page 29, Section 13	Added paragraph below about the Statistical Analysis Plan developed for the study.<br>A separate document, the *Statistical Analysis Plan*, provides a detailed and comprehensive description of the statistical approach to address the various study objectives and the statistical considerations for analysis of the study data. This *Statistical Analysis Plan* supersedes the following section of the protocol that provided an initial descriptions of the analysis plans. The *Statistical Analysis Plan* will be locked (fixed) prior to the final closure of the study data base after the completion of follow-up of the study cohort.
Page 37, Section 15.8	Deleted April
Page 39, References Cited	Reference 15 updated for newer reference: 2017 Standards of Care in Diabetes.

TABLE OF CONTENTS

1.0 PROJECT SUMMARY 1
2.0 BACKGROUND AND SIGNIFICANCE 3
3.0 SPECIFIC AIMS 4
4.0 RESEARCH DESIGN 5
Figure 1. Patient Enrollment and Study Design 6
Figure 2. Metabolic outcomes and subsequent therapy 7
5.0 INTERVENTIONS 8
5.1 Run-in and Metformin Initiation and/or Titration 9
5.2 Randomization to One of Four Medications (all study medications to be provided free-of-charge to participants) 9
5.3 Management of Glycemia 11
5.3.1 Diabetes Management after Primary Outcome Has Been Reached 12
5.3.2 Diabetes Management after Secondary Metabolic Outcome Has Been Reached 12
5.3.3 Diabetes Management after Tertiary Metabolic Outcome Has Been Reached 12
5.4 Other (Non-Diabetes) Interventions 12
6.0 STUDY POPULATION 13
6.1 Inclusion Criteria 13
6.2 Exclusion Criteria 14
7.0 RECRUITMENT 15
7.1 Screening 16
7.2 Run-in 16
7.3 Randomization 16
Table 1: Run-in Schedule 17
8.0 OUTCOME ASSESSMENTS 18
8.1 Protocol Outcome Measurements and Assessments 18
8.2 Other Metabolic Measures (for phenotyping and determination of mechanisms of success or failure of interventions) 19
Table 2: Schedule of Measurements and Assessments 21
9.0 STANDARD DIABETES EDUCATION 22
10.0 ADVERSE EVENT REPORTING 22
10.1 Serious Adverse Event Reporting 23
10.2 Other Adverse Events 23
11.0 OTHER MEASUREMENTS AND ANCILLARY STUDIES 23
12.0 STUDY OUTCOMES 23
12.1 Primary Outcome 23
12.2 Other Metabolic Outcomes 24
12.3 Health-Economic Evaluation 24
12.4 Secondary Composite Outcomes 24
12.5 Microvascular and Cardiovascular Outcomes 26
12.6 Other Outcomes (Table 2 lists the measurement frequency for outcomes) 27
13.0 STATISTICAL ANALYSIS PLANS 29
13.1 General Analysis Strategies 29
13.2 Primary Outcome 29
13.3 Other Outcomes 30
13.4 Composite Outcomes 31
13.5 Interim Analyses 31
13.6 Subgroup and Stratified Analyses 31
14.0 SAMPLE SIZE AND POWER 32
14.1 Primary Outcome 33
14.2 Secondary Outcomes – Microalbuminuria and Clinical Cardiovascular Disease 34
14.3 Subgroup Analyses 34
15.0 POLICIES, ETHICAL CONSIDERATIONS, AND COMPLIANCE 35

15.1 IRB Approval 35
15.2 Protocol Changes 35
15.3 Participating Clinical Sites 35
15.4 Informed Consent 35
15.5 Study Subject Confidentiality 36
15.6 Sample and Data Storage 36
15.7 Preservation of the Integrity of the Study 36
15.8 Study Timeline 37
16.0 STUDY ADMINISTRATION 37
16.1 Study Leadership and Governance 37
16.2 Subcommittees 37
16.3 Coordinating Center and Chairman's Office 38
16.4 Data and Safety Monitoring Board 38
BIBLIOGRAPHY AND REFERENCES CITED 39

1.0 PROJECT SUMMARY

The epidemic of type 2 diabetes that has affected the US and other populations in the last half of the 20th and first part of the 21st centuries threatens to become the major public health problem of this century, affecting up to 1 in 3 Americans if current trends continue. The most recent estimates in the US include a prevalence of more than 24.5 million persons with type 2 diabetes, with an incidence of 1.9 million new cases per year. The major human and economic costs associated with the epidemic are related primarily to the development of long-term complications including retinopathy, nephropathy, and neuropathy that cause more cases of blindness, renal failure, and amputations than any other disease. Cardiovascular disease is increased 2-5 fold in type 2 diabetes and is the leading cause of premature death. High quality clinical trials have established the importance of lowering glycemia with a variety of medications to reduce the long-term complications. One of the major challenges for practitioners is to choose, from the considerable armamentarium of glucose-lowering medications at their disposal, the optimal approach to achieving and then maintaining good glycemic control for as long as possible. Evidence supporting the choice of one versus another agent as initial therapy or as the second drug added to metformin, the consensus initial treatment for type 2 diabetes, is lacking. Comparative effectiveness research is a high priority both to improve public health and to maximize cost-effectiveness in the management of type 2 diabetes. Moreover, efforts to individualize therapies and determine whether some therapies work better in individuals with particular characteristics compared to others are needed, and the differential effects of various therapies on the physiology of glucose metabolism also remain unknown.

The **G**lycemia **R**eduction **A**pproaches in **D**iabetes: A Comparative **E**ffectiveness (GRADE) Study will address these questions in a randomized clinical trial in patients with <10 years duration of diagnosed type 2 diabetes that will compare the metabolic effects of four common anti-diabetic drugs when combined with metformin. The four randomly assigned medications are the sulfonylurea glimepiride, the DPP-4 inhibitor sitagliptin, the GLP-1 agonist liraglutide, and the basal insulin glargine. A total of up to 5000 patients who are within 10 years of diagnosis and are being treated with metformin at the time of recruitment will be enrolled. These subjects will be randomly assigned to one of four agents, which will be added to metformin, to compare the effects among these four combinations. The proposed study compares four of the major diabetes drug combinations over a clinically meaningful duration, with a possible mean follow-up of 4.8 years, allowing for a lag in recruitment and losses to follow-up.

The *primary metabolic outcome* will be the time to primary failure defined as a hemoglobin A1c (HbA1c) $\geq$7%, subsequently confirmed, after having been treated with maximally tolerated doses of both metformin (up to 2000 mg per day) and the randomly assigned second medication (intention-to-treat). A *secondary metabolic outcome* will be the time to an HbA1c >7.5%, confirmed. At that time, basal insulin “rescue” therapy will be added for the subjects assigned to drugs other than insulin, and insulin therapy will be intensified for those assigned to basal insulin. Another metabolic outcome is the time to tertiary metabolic failure defined as the time to another HbA1c >7.5%, confirmed, after treatment with basal insulin, at which time more intensive insulin therapy is initiated. Other metabolic outcomes to be studied include the mean HbA1c and measures of insulin secretion and resistance over the entire study duration and at intermediate time points. Other important attributes of the four drug combinations to be assessed include adverse effects such as weight gain and hypoglycemia, effects on cardiovascular disease (CVD) risk factors, tolerability and quality-of-life, and cost and cost-effectiveness. Although the GRADE study is not primarily a clinical outcomes study, selected measures of microvascular disease will be included as secondary outcomes and

clinical cardiovascular outcomes will be recorded. We will also examine the phenotypic and, resources permitting, the genotypic characteristics that are associated with metabolic response to and/or failure of the individual medication combinations. Mechanistic studies of the pathophysiology contributing to success or failure of individual combinations will be part of the trial.

When basal insulin (glargine) is added to metformin and the assigned medication at the time of secondary metabolic failure in participants who were originally assigned to medications other than insulin, the same insulin adjustment protocol as implemented for the participants originally assigned to glargine will be used. If tertiary metabolic failure then occurs, metformin and basal (glargine insulin) will be continued, the originally assigned medication will be discontinued, and the insulin regimen will be intensified with the addition of rapid-acting insulin according to the study insulin protocol.

Similarly, if the participants originally assigned to basal insulin reach the secondary metabolic outcome following the primary outcome, their metformin and basal insulin will be continued and the insulin regimen will be intensified. The systematic, study-wide implementation of intensified ("rescue") insulin therapy will allow the study to determine the relative effectiveness of the four assigned therapies to affect the time period until intensified insulin therapy (basal insulin plus rapid-acting insulin) is necessary.

The major specific aims of this clinical trial in metformin-treated patients with <10 years duration of diagnosed type 2 diabetes include:

- Comparison of the relative effectiveness of four commonly used glucose-lowering medications with different glucose lowering mechanisms, when used in combination with metformin, in maintaining metabolic control, defined as time-to-primary failure with an HbA1c ≥7.0%, confirmed, while on maximally tolerated doses of both metformin, up to 2000 mg per day, and the assigned medications;
- Comparison of the relative attributes, including the durability of the glycemia-lowering effects and other metabolic outcomes, adverse effects, effects on CVD risk factors and quality-of-life, tolerability and cost-effectiveness, of the four glucose-lowering medications used in combination with metformin;
- Comparison of the cumulative incidence of diabetic complications, such as microalbuminuria, among the randomly assigned agents;
- Determination of the phenotypic characteristics associated with response to and failure of the four different medication combinations;
- Evaluation of factors that determine the success and/or failure of specific regimens over time, including mechanistic studies of beta-cell failure/preservation over time;
- Determination of the relative effects of the four combinations on the time to secondary metabolic failure, with an HbA1c >7.5%, confirmed, requiring the need for rescue therapy;
- Determination of the relative effects of the four combinations on the time to the need to implement intensive insulin therapy with basal plus rapid-acting insulin.

The results of this trial will identify the most effective means of treating type 2 diabetes and will have major public health implications.

2.0 BACKGROUND AND SIGNIFICANCE

The epidemic of type 2 diabetes that has affected the US and other populations in the last half of the 20th and first part of the 21st centuries threatens to become the major public health problem of this century, affecting up to 1 in 3 Americans if current trends continue (1). The most recent estimates in the US include a prevalence of more than 24.5 million persons with type 2 diabetes, with an incidence of 1.9 million new cases per year (1). The major human and economic costs associated with the epidemic are related primarily to the development of long-term complications including retinopathy, nephropathy, and neuropathy that cause more cases of blindness, renal failure, and amputations than any other disease (2) in the developed world. Cardiovascular disease (CVD) is increased by 2-5 fold in type 2 diabetes and is the leading cause of death and premature death in persons with diabetes (3). The estimated annual cost of diabetes in the US in 2007 was $174 billion dollars per year with the greatest direct cost ($58 billion) related to its chronic complications (4). The annual expenditure for glucose-lowering drugs in the US in 2007 was $13 billion, having almost doubled since 2001 (5).

There are several reasons for guarded optimism in the setting of this ongoing epidemic, which is associated with the relentless increase in obesity. First, clinical trials have demonstrated effective means of delaying or preventing the development of diabetes. If these interventions were implemented successfully, they could decrease the annual incidence of diabetes by as much as 58% (6-8). Second, high quality clinical trials have shown that intensive management of glycemia, especially when instituted early after diagnosis, can substantially reduce the long-term microvascular, neuropathic, and cardiovascular complications (9-11). Third, in the past decade, the diabetes epidemic has spurred the development of seven new classes of glucose-lowering medications that may contribute to our ability to control glycemia more effectively and thus reduce complications, although no single agent or combination of agents has been shown to be superior in the long term (12). Finally, numerous high-quality clinical trials have shown that blood pressure- and lipid-lowering medications can reduce CVD in type 2 diabetes as effectively as they do in the non-diabetic population (13) and that CVD risk in diabetes is decreasing (14).

One of the major challenges for practitioners is to choose, from the considerable armamentarium of glucose-lowering medications at their disposal, the best means for achieving from the outset and then maintaining an appropriate level of glycemic control over time. European and US evidence-based consensus algorithms have been developed with the goal of helping clinicians to select among the numerous medications, and their combinations, to achieve and maintain a target HbA1c level less than 7% (15-17). Other published algorithms have selected different glycemic goals and recommended different strategies to achieve them (18). Unfortunately, the development of all of these algorithms and recommendations has been hampered by the dearth of long-term head-to-head comparator studies of different glucose-lowering medications, either alone or in combinations. This is especially true of the newer, brand-name medications. Moreover, only a few studies have examined the durable effects of interventions on glycemic control (10, 19, 20), with the vast majority of drug trials lasting from only 3 to 12 months. Since type 2 diabetes is a progressive disease with worsening metabolic control over time, the long-term effects of interventions are of particular importance. Safety, side-effect profiles, tolerability, patient acceptance, burden of therapy, and cost are all-important factors in the chronic treatment of this long-term degenerative disease. Finally, recent position statements have emphasized individualization and “patient-centered” approaches to therapy (16), but there are few studies that provide relevant insights into which patients might do better or worse with specific therapies.

Given the importance of achieving and maintaining adequate glycemic control over time, it is critical to understand the relative effectiveness of the different medications and their combinations in maintaining glycemic goals over time. The tolerability, adverse effects, impact on cardiovascular risk factors, and cost of the commonly used medication combinations represent important characteristics that also need to be compared and considered in the choice of interventions. Comparative effectiveness research has recently been identified as a high national priority in the US (21). Similarly, improved understanding of phenotypic, physiologic, and perhaps genotypic differences between patients that affect responses to one medication or combination of medications over others has been identified as a critical element in individualizing therapy for maximum effectiveness (22). Notably, most pharmaceutical company supported studies have failed to address either comparative effectiveness over time or inter-patient differences that may affect responses to therapy. As a result, patients with type 2 diabetes are currently treated similarly, without taking into account individual characteristics that might direct the choice of more effective interventions.

These major aims, focusing on a comparison of the effectiveness and other clinically important attributes of glucose-lowering medications, have major health economic implications, in addition to their obvious public health impact. The cost of glucose-lowering medications has doubled from 6.3% of all prescribed drug spending in 2004 to 12.2% in 2006 with unacceptable costs per QALY based on currently available data for at least some of the new medications (5).

3.0 SPECIFIC AIMS

The major specific aims of this comparative effectiveness clinical trial in metformin-treated patients with <10 years duration of diagnosed type 2 diabetes include:

- Comparison of the relative effectiveness of four commonly used glucose-lowering medications with different mechanisms of action, when added to metformin, in maintaining metabolic control, defined as time-to-primary failure with an HbA1c ≥7.0%, confirmed, while on maximally tolerated doses of both metformin, up to 2000 mg per day, and the assigned medication;
- Comparison of the relative attributes of the four glucose-lowering medications used in combination with metformin on the durability of the glycemia-lowering effects and other metabolic outcomes, adverse effects, effects on CVD risk factors and quality-of-life, tolerability and cost-effectiveness;
- Comparison of the cumulative incidence of diabetic complications, such as microalbuminuria, among the four agents under study;
- Comparison among the four agents of the cumulative incidence of major cardiovascular disease
- Comparison among the four agents of the incidence of cardiovascular abnormalities as detected by ECG
- Determination of the phenotypic characteristics associated with response to and failure of the four different medication combinations;
- Evaluation of factors that determine the success and/or failure of specific regimens over time, including mechanistic investigations of beta-cell function over time;
- Determination of the relative effects of the four combinations on the time to secondary metabolic failure, with an HbA1c >7.5%, confirmed, requiring the need for rescue therapy;
- Determination of the relative effects of the four combinations on the time to the need to implement intensive insulin therapy with basal plus rapid-acting insulin following

tertiary metabolic failure (HbA1c >7.5% confirmed, while treated with metformin, the original assigned medication, and basal insulin) among those not originally assigned to basal insulin, or secondary failure among those assigned to basal insulin.

4.0 RESEARCH DESIGN

This parallel group, unmasked clinical trial will randomize up to 5000 consenting subjects with <10 years duration of diagnosed type 2 diabetes, HbA1c from 6.8-8.5%, and who have been treated with metformin alone (**Figure 1**). Subjects will adjust metformin during run-in, as necessary, aiming for 2000 mg per day and those unable to tolerate at least 1000 mg/day will be ineligible.

Eligible participants will be randomly assigned to one of four glucose-lowering medications shown in Figure 1 in combination with metformin. The principal comparisons will be among the four drug groups starting from the time of randomization.

The primary and secondary outcomes are reviewed in detail in Section 12. Briefly, the primary outcome is the time to the observation of an HbA1c ≥7%, subsequently confirmed while receiving the maximally tolerated dose of the assigned regimen (intention-to-treat principle). The secondary outcome is the time to the observation of an HbA1c >7.5%, subsequently confirmed, and the tertiary outcome is defined as the time to another HbA1c >7.5%, confirmed, after treatment with basal insulin, at which time an intensive basal/bolus insulin regimen is initiated. Each of these outcomes is counted while receiving the maximally tolerated dose of the assigned regimen and regardless of adherence to assigned medications at the time of the HbA1c test according to principles of intention-to-treat analysis.

The proposed study design and recruitment plan will allow a practical head-to-head comparison among four different therapy combinations. The trial is designed to be pragmatic (i.e. with immediate potential for translation) since we will be using approved medications and their combinations according to labeling.

Figure 1. Patient Enrollment and Study Design

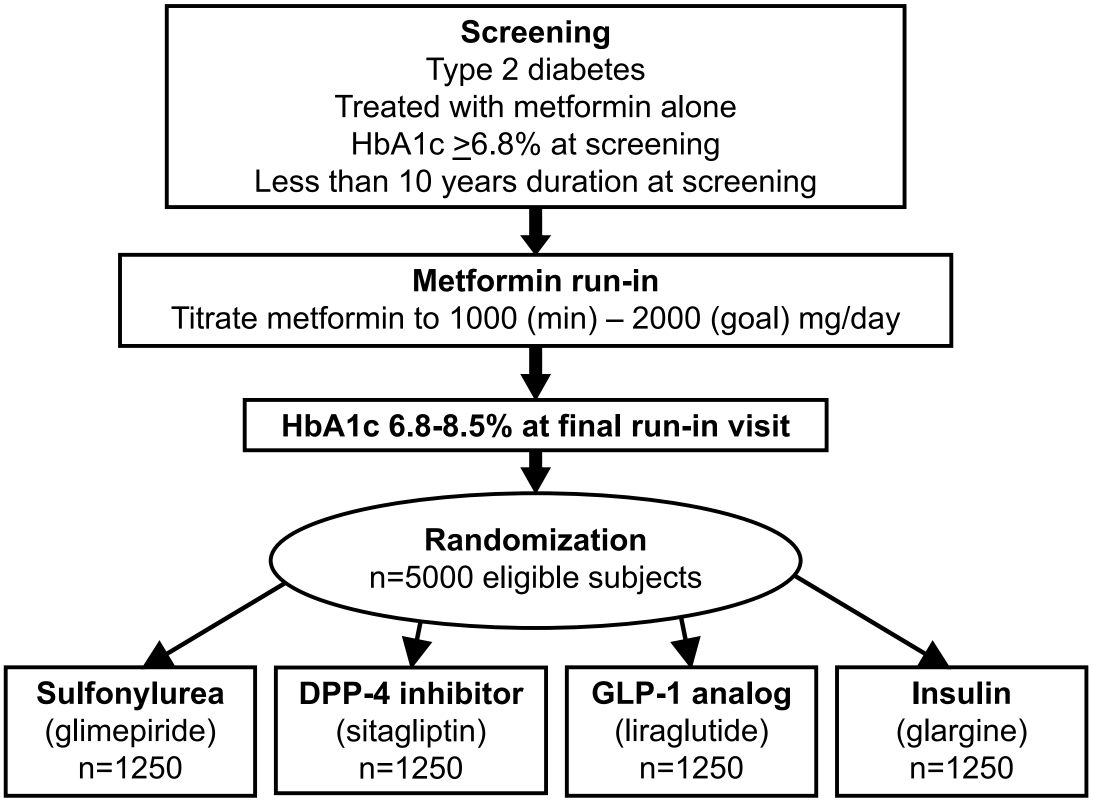

The trial will be conducted under an intent-to-treat design such that all randomized subjects will continue follow-up and complete all outcome assessments until the planned conclusion of the study (planned follow-up of approximately 4 to 7.5 years, depending on the time of entry), including those who have reached the primary outcome. Otherwise, analyses of all outcomes would be susceptible to a healthy survivor effect where the only subjects evaluated at out years would be those who have not yet experienced primary failure of the assigned regimen.

In order to encourage retention in the study over time and ensure a longer exposure to the study drug combination for the purposes of intention-to-treat analyses of other outcomes, such as microalbuminuria, assigned study medications will be continued until the need for intensification of insulin therapy with basal plus rapid-acting insulin (**Figure 2**). At the time that the secondary metabolic outcome occurs, participants who were assigned to study medications other than insulin will have basal insulin added to continued metformin and the original randomly assigned second medications.

Figure 2. Metabolic outcomes and subsequent therapy

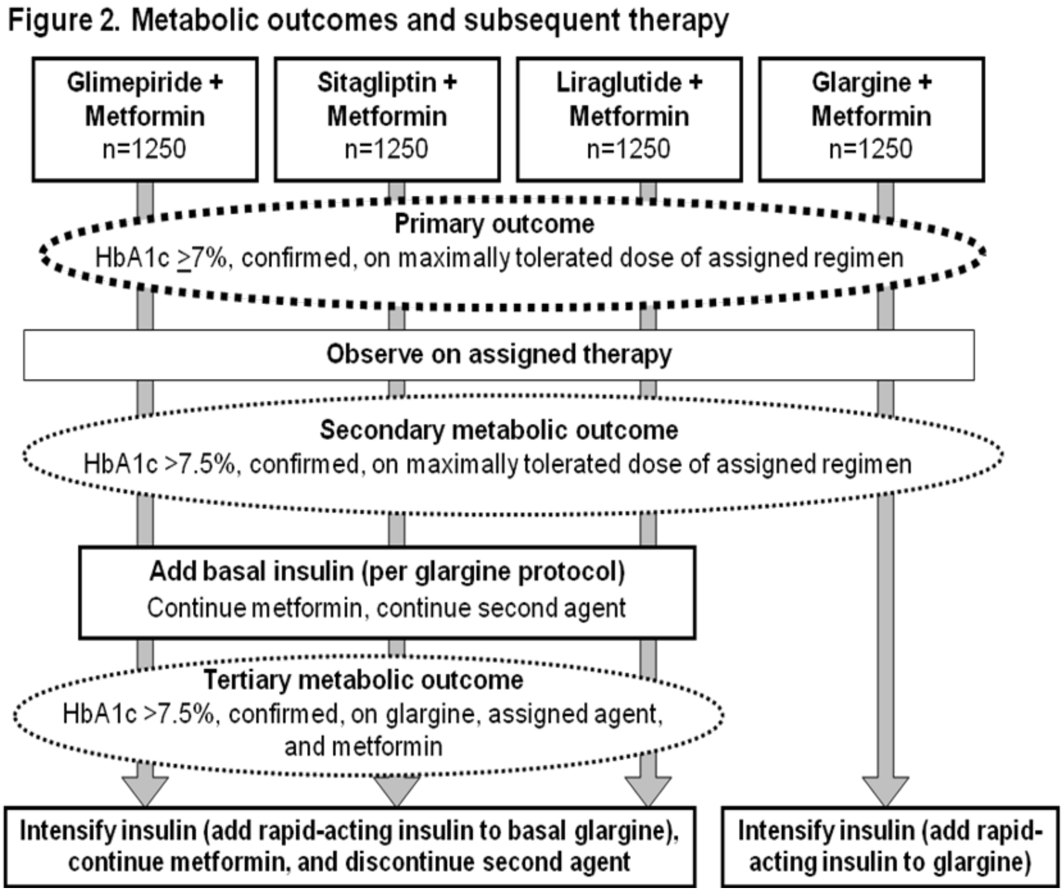

The basal insulin will be adjusted according to the study insulin protocol (see Section 5.2). Any subject whose HbA1c again reaches >7.5%, confirmed, while treated with basal insulin (after secondary metabolic outcome) will be considered to have reached the tertiary metabolic outcome. These subjects will continue their metformin and basal insulin therapy regimen, intensify the insulin regimen with the addition of rapid-acting insulin, according to study guidelines, and discontinue the original randomly assigned medications.

Among subjects assigned to basal insulin plus metformin, when the secondary metabolic outcome occurs, the insulin regimen (with metformin) will likewise be intensified. Since the glargine-assigned group is already treated with glargine, they will essentially reach their secondary and tertiary outcome simultaneously.

All subjects will be followed in GRADE until study end. Metformin, the randomly assigned drugs, and insulin and other supplies required for study insulin initiation, adjustment, and intensification will be supplied free-of-charge throughout the study.

5.0 INTERVENTIONS

Metformin has been selected as the foundation therapy based on the same rationale and characteristics as were used in the recently developed consensus algorithm (17): namely, its long-term clinical experience; effectiveness in lowering glycemia over a wide range of HbA1c levels without causing hypoglycemia; its weight-neutral or weight-loss effect; probable cardiovascular risk reduction (10, 11, 23); safety profile; side-effect profile; high level of patient tolerance; and its low cost. Recent studies have shown that a large majority of patients with recent-onset T2DM are treated with metformin, making this design both practical and clinically relevant. Metformin treatment is associated with an increased risk of vitamin B12 deficiency (15).

At the time of randomization, all subjects will be randomly assigned to one medication in each of the following classes to be added to metformin (**Figure 2**):

- Sulfonylurea: glimepiride
- DPP-4 inhibitor: sitagliptin
- GLP-1 receptor agonist: liraglutide
- Insulin: glargine

Given the complexity of the trial and a desire to use a pragmatic design, the interventions will not be masked.

The classes of medications to be studied in combination with metformin comprise the majority of glucose-lowering medications prescribed in the US. This study provides an opportunity to evaluate systematically the benefits of clinically relevant combinations and is unique in performing head-to-head comparisons of as many brand name and generic drug combinations as permitted by the available resources over a prolonged period.

The selection of medications and their combinations was predicated on the most commonly used approved combinations and the availability of at least some preliminary data to support the glycemia-lowering effectiveness, safety, and tolerability of the combinations. Of the nine classes of agents available in addition to metformin, we propose to study four of the most commonly prescribed in the U.S. Increasing concern regarding pioglitazone's future, owing to the putative increased risk for bladder cancer (24), superimposed on previously established volume retention and bone loss, contributed to its elimination from the study. The potential adverse impact on recruitment of including a drug that is receiving increasing and highly visible negative attention and the lack of donated or affordable pioglitazone were additional considerations. Since the four drug classes proposed capture the majority of glucose-lowering drugs used, the study will be clinically relevant and its results generalizable (5). All of the four combinations have been approved by the FDA and its European and Canadian counterparts, making the results of the trial immediately and widely translatable to practice.

We have proposed specific agents within the four classes, all of which are FDA-approved in their proposed initial combinations, dictated by their specific attributes. The criteria by which specific agents were chosen within classes included differences between the agents in the following: lowering of glycemia, published side-effect profiles, effects on CVD risk factors, clinical experience, and ease of administration and acceptability. In cases where there were no appreciable or substantive differences between agents within the classes, consideration was

given to those agents that are used most frequently and were made available by the manufacturers.

After the primary metabolic outcome and secondary metabolic outcome have been reached, the combination of metformin and randomly assigned medication will be continued (**Figure 2**) as basal insulin is added. The rationale for the continued combination therapy is to enable the further study of which combination(s) may delay the further metabolic worsening to the need for insulin intensification, another metabolic outcome. Moreover, the use of three agents is becoming increasing popular in routine clinical practice.

5.1 Run-in and Metformin Initiation and/or Titration

All subjects who meet eligibility criteria following the screening visit will enter a run-in period (minimum approximately 6 weeks, maximum approximately 14 weeks from the time of screening to baseline visit, depending on metformin exposure prior to screening and rate of adjustment during run-in) (see Section 7.2). Potential subjects who are at or above the study-specified maximum dose of metformin (2000 mg per day), either immediate- (IR) or extended-release (XR), can complete the run-in visits (initial to final run-in visits) in as little as 4 weeks, whereas potential subjects who need to increase metformin to achieve the study-specified dose can complete the run-in in as little as 6-8 weeks. The goals of the run-in period include the following: adjustment of metformin dose to a goal of 2000 mg daily (minimum 1000 mg daily); and demonstration of adherence to study procedures (visit attendance, medication taking) and of willingness to perform self-monitoring of blood glucose and self-injection. Before the end of the run-in, all subjects will receive standardized diabetes education, which will provide basic knowledge about T2DM, including skills and behaviors that are important for successful management such as medication taking, healthy eating and weight loss, increased physical activity, and smoking cessation. Diabetes education that provides diabetes support and education consistent with the aims of the Look AHEAD (Action for Health in Diabetes) program (20) and/or designed specifically for the GRADE study will be used.

Metformin will be adjusted as follows. During the run-in period, all participants will be changed to study supplied metformin. If already taking 2000 mg per day of metformin or metformin XR and tolerating that dose, no further changes will be made. If taking more than 2000 mg per day of metformin, their dose will be reduced to 2000 mg per day. If taking less than 2000 mg per day, the potential participants will increase the dose approximately weekly by 500 mg, with doses taken with meals (usually breakfast and dinner) to a target dose of 2000 mg daily, as tolerated. If potential participants develop gastrointestinal intolerance during metformin IR titration, metformin XR can be used to try to get them to 2000 mg daily or to a minimum of 1000 mg daily. Potential participants who do not tolerate at least 1000 mg per day of metformin will not be eligible for randomization. Metformin IR or XR will be provided free-of-charge to study participants. Subjects who enter run-in but are not randomized will be provided with study metformin as they transition their diabetes care back to their primary care provider.

5.2 Randomization to One of Four Medications (all study medications to be provided free-of-charge to participants)

Eligible subjects who tolerate at least 1000 mg per day of metformin during run-in will be randomly assigned to one of the four other glucose-lowering medications, which will be added to metformin at the time of randomization. Glucose treatment decisions will be driven by protocol and controlled solely by the study staff. Self-monitoring of blood glucose (SMBG) will differ by medication class and may be individualized by study clinical staff. Medications that are titrated

in usual care and consistent with their labeling will be titrated to achieve fasting glucose levels 80-130 mg/dl based on self-monitoring of blood glucose without symptomatic hypoglycemia or to the maximum tolerated dose, whichever dose is lower.

In the ACCORD trial, polypharmacy (with as many as five drugs and with 62% of the intensive treatment group requiring $\geq$3 agents) aiming for an HbA1c <6.0% and achieving a median HbA1c of 6.4% were shown to be associated with a significantly increased risk of total and CVD mortality, especially in subjects with longer diabetes duration and pre-existing CVD (25). The increased risk did not appear to be associated with hypoglycemia (26) or with the reduction in HbA1c; the increased risk was greatest among those who did not reduce their HbA1c (27). The GRADE cohort and intervention strategies and goals are not analogous, because subjects are required to have <10 years duration of diagnosed diabetes and will be prescribed no more than two agents as part of the protocol until the secondary metabolic outcome is reached. For the relatively small fraction of non-insulin assigned subjects who reach the secondary metabolic outcome, a maximum of three drugs will be used. Therefore, no adjustments in therapy will be made for HbA1c levels <6.5% unless subjects have symptomatic hypoglycemia or other dose-related side effects. In that setting, doses of medications responsible will be reduced.

Sulfonylureas: Glimepiride will be used as the study sulfonylurea, giving preference to a sulfonylurea with lower risk for hypoglycemia (28). Participants will be started at 1 or 2 mg once daily, based on baseline HbA1c, and increased weekly based on self-monitored fasting glucose measurements to a maximum dose of 8 mg, assuming that hypoglycemia (see Section 8.1) has not occurred. SMBG will be mandated during the titration phase and at least twice weekly thereafter.

DPP-4 inhibitors: There are currently three approved DPP-4 inhibitors in the US (29-31). Sitagliptin 100 mg once per day has been selected (with dose adjustment based on renal function). Unlike several other DPP-4 inhibitors, sitagliptin does not have interactions with other drugs due to CYP enzyme inhibition. No titration is necessary.

GLP-1 receptor agonists: Exenatide twice daily and once weekly (the LAR formulation), and liraglutide once daily are the only currently approved GLP-1 receptor agonists in the US (32-34). We have chosen liraglutide in part for its once-per-day convenience. A multi-dose pen will be used to initiate therapy at 0.6 mg once-per-day. The dose will be advanced to 1.8 mg during the first month, assuming that intolerable gastrointestinal or other side effects do not occur.

Basal insulins: Glargine insulin has been selected as the basal insulin, primarily because of ease of once-per-day administration, minimal hypoglycemia, and well-established algorithms to titrate doses. Glargine will be initiated with a dose of up to 20 units daily and dose will be adjusted based on an algorithm modified from the INSIGHT protocol (35) aiming for a fasting glucose of 80-130 mg/dl without hypoglycemia, consistent with current American Diabetes Association guidelines (15). SMBG will be mandated during the titration phase and at least twice weekly thereafter.

Other classes: Other classes of glucose-lowering drugs in addition to pioglitazone (discussed above) and including the alpha-glucosidase inhibitors (acarbose) (36), non-sulfonylurea sulfonylurea receptor agonists (nateglinide and repaglinide) (37), rapid-acting insulins (38), the bile acid sequestrant colesevelam (39), and dopamine agonist bromocriptine (40), were not selected for the study based on a number of considerations including potential safety concerns,

limited clinical use and experience in new onset type 2 diabetes, and/or relatively low efficacy, poor tolerability and frequent side-effects.

5.3 Management of Glycemia

Study staff will communicate directly with participants' primary care providers (PCPs) so that it is clear that glycemic management will be assumed by the study staff through the entire course of the study. Similar strategies in the Diabetes Control and Complications Trial (DCCT), United Kingdom Prospective Diabetes Study (UKPDS) and in the ongoing Look AHEAD study resulted in almost no interference with the study implemented interventions and have promoted good retention (9, 10, 20). Subjects will be seen quarterly. Glycemic management for participants assigned to glimepiride or insulin will follow an established titration protocol based on self-monitoring of blood glucose, aiming for fasting glucose between 80 and 130 mg/dl without symptomatic hypoglycemia. Additionally, medications will be titrated based on HbA1c values ≥7.0%, up to the maximally tolerated dose. Reminders of other ADA-recommended care and surveillance for complications will be provided to the patients' primary care providers on a regular basis (15). Results of clinically relevant testing performed as part of GRADE will be forwarded to subjects' primary care providers.

In the event that a participant fails to achieve acceptable glycemic control, based on a sustained HbA1c > 8.5% for two measurements after being eligible to start insulin, the GRADE treatment team will contact the participant's PCP to discuss whether non-GRADE medications might be indicated. The decision to add non-study drug(s) and their prescription will be left to the PCP. GRADE medications, and glucose monitoring equipment/supplies, as per protocol, and all study assessments and follow-up will continue to be supplied free-of-charge.

Although consideration was given to masking HbA1c values, in order to limit potential bias in the application of the medications and treatment strategies in this unmasked trial, we chose to share HbA1c values with the participants as it more closely resembles usual care. Moreover, although glucose treatment decisions will be driven by protocol and controlled solely by the study staff, patients are likely to have HbA1c levels drawn outside of the study, so that masking of levels would be impractical. Adherence to medications and to recommended lifestyle interventions (dietary, activity level, and smoking cessation) will be reinforced for all subjects at visits.

Vitamin B12 levels will be monitored at intervals during the study because of the small, but increased, risk for B12 deficiency in metformin treated patients (15). Self-monitoring of blood glucose (SMBG) will be performed for safety (to prevent hypoglycemia) on a specified schedule for participants assigned to insulin or sulfonylurea according to usual care recommendations (15). Medications (insulin and sulfonylureas) will be titrated between visits based on results of SMBG as is common in the course of usual care. SMBG will also be performed for safety reasons by all participants in the presence of symptoms that suggest hypoglycemia, hyperglycemia, or during inter-current illness likely to affect glucose control substantially.

Female participants who become pregnant during the study will be referred back to their PCP with the recommendation that the subject be referred to a high-risk obstetrical team. Patients will be transitioned rapidly to non-study medications (PCP and obstetrical management team) with the goal of stopping the study medication and starting the drug regimen recommended for pregnancy as soon as possible. Study medications will be restarted in the post-partum period after breastfeeding has stopped.

All participants will be followed in GRADE until study end. All participants will continue to return for quarterly outcome measurements until study end. The clinically relevant measurements such as HbA1c, blood pressure, and lipid levels, will continue to be shared with the subjects' health care provider. The continuation of assigned therapies until the tertiary metabolic outcome is reached and insulin is intensified will facilitate the study of the differences in long-term effects of the four medications on secondary outcomes.

5.3.1 Diabetes Management after Primary Outcome Has Been Reached

All participants, including those who have reached the primary and/or secondary outcomes, will continue to be followed until the designated study end-date has been reached, under the intention-to-treat principle. After the primary outcome (HbA1c $\geq$7%, confirmed) has been reached, randomly assigned study drugs and metformin will be continued and supplied free-of-charge. This will continue until a secondary metabolic outcome of HbA1c >7.5%, confirmed similarly to the primary outcome (with confirmation at the next 3-month visit unless HbA1c is >9% in which case confirmation is performed at 3 to 6 weeks) occurs.

5.3.2 Diabetes Management after Secondary Metabolic Outcome Has Been Reached

<u>Participants assigned to agents other than insulin</u>

At the time that the secondary metabolic outcome occurs, participants will have basal insulin added to continued metformin and their randomly assigned study medications. The basal insulin will be adjusted according to the study insulin protocol (see Section 5.2). If, after being started on basal insulin, the participant's HbA1c reaches or remains >7.5%, confirmed, they will be considered to have reached the tertiary metabolic outcome. At that time, intensive insulin therapy will be initiated.

<u>Participants assigned to basal insulin</u>

At the time that the secondary metabolic outcome occurs, the participants originally assigned to basal insulin will initiate intensive insulin therapy with the addition of rapid-acting insulin to metformin and basal insulin.

5.3.3 Diabetes Management after Tertiary Metabolic Outcome Has Been Reached

<u>Participants assigned to agents other than insulin</u>

Any participants originally assigned to an agent other than insulin who have reached the tertiary metabolic outcome (HbA1c >7.5%, confirmed, while treated with metformin, assigned medication and basal insulin) will have their assigned medication stopped and insulin regimen intensified with the addition of rapid-acting insulin. The additional rapid-acting insulin injection(s) will be adjusted by the clinic staff based on the study insulin protocol.

5.4 Other (Non-Diabetes) Interventions

Other than the diabetes interventions that are the focus of this trial, all other treatments, including the management of hypertension and dyslipidemia, will be performed by the participant's own health care provider. We will provide references to the American Diabetes Association (ADA) guidelines to all clinicians providing care to the participants (15). Moreover, clinical alerts will be established for those clinically relevant outcomes that are measured as part of the study. Blood pressure will be measured at regularly scheduled study visits using standardized methods. Fasting lipoprotein levels will be measured periodically. Medication history will also be collected quarterly. The levels of CVD risk factors, events, and their treatments will be secondary outcomes with the goal of determining differential effects of the randomly assigned diabetes medication combinations, adjusting for treatment of co-incident conditions.

6.0 STUDY POPULATION

Addendum 08/24/16.

The original protocol sample size target of 5000 subjects provided excellent power for the primary and key secondary outcomes. Calculations assumed that the 5000 subjects would be enrolled over a 3 year period and the total study treatment and follow-up would be 7 years. Owing to an approximate 6 month lag in recruitment, recruitment has been extended by approximately 6 months to end in summer 2017. While we hope to achieve a sample size of 5000, current recruitment trends suggest a sample size of about 4800 will be achieved. To allow for the loss in power, the period of follow-up will also be increased by 6 months. Additional power calculations show that the 7.5 years of follow-up with a sample size of 4800 will preserve the original excellent levels of power. Since the final number to be enrolled is unknown, we now state that 4800-5000 patients will be enrolled. However, the original calculations of study power remain unchanged assuming 5000 subjects enrolled under the original design assumptions.

Through an open competition process a total of 37 clinical centers in the United States have been selected, each of which will aim to enroll about 150 (up to 250) eligible subjects to reach the study-wide total enrollment of 4800-5000 subjects over a period of approximately 3.5 years. Individual site recruitment targets may be increased or decreased based on performance and study needs.

The study will recruit and follow people with type 2 diabetes with less than ten years since diagnosis at the time of screening. The majority of potential subjects will be identified based on a prior diagnosis of diabetes detected through reviews of medical histories and self-report and aided by use of electronic medical records and other databases. In order to be eligible, potential subjects must have an HbA1c of 6.8-8.5%, at final run-in visit (~2 weeks prior to randomization), measured in the central laboratory.

The study population (**Figure 1**) of up to 5000 participants will include patients with <10 years duration of diagnosed type 2 diabetes previously treated with metformin and on no other glucose-lowering medications.

We will aim to recruit as much representation as possible from racial and ethnic minority groups that are disproportionately affected by type 2 diabetes, encouraging the clinical centers to pay particular attention to these populations during recruitment. Similarly, we will try to recruit a substantial fraction (e.g. >20%) who are 60 years of age and older. Clinical centers were selected to ensure an adequate distribution by race/ethnicity and age. Specific participation criteria are noted below.

6.1 INCLUSION CRITERIA

1. Men or women ≥30 years of age at time of diabetes diagnosis; for American Indians, age is ≥20 years at time of diagnosis
2. Duration of diagnosed diabetes <10 years determined as accurately as possible based on available records at screening
3. HbA1c criteria (at final run-in visit, ~2 weeks prior to randomization): 6.8-8.5%
4. Taking a daily dose of ≥1000 mg metformin for a minimum of 8 weeks at final run-in

5. Willingness to administer daily subcutaneous injections, take a second glucose-lowering drug after randomization, potentially initiate insulin and intensify insulin therapy if study metabolic goals are not met, and perform self-monitoring of blood glucose
6. Fluent in either English or Spanish
7. A negative pregnancy test for all females of childbearing potential (i.e. pre-menopausal, and not surgically sterile)
8. Provision of signed and dated informed consent prior to any study procedures

6.2 Exclusion Criteria

1. Suspected type 1 diabetes (lean with polyuria, polydipsia, and weight loss with little response to metformin) or "secondary" diabetes due to specific causes (e.g. previously diagnosed monogenic syndromes, pancreatic surgery, pancreatitis)
2. Current or previous (within past 6 months) treatment with any diabetes drug/glucose-lowering medication other than metformin (limited use of no longer than seven days is allowed, for example during hospitalization)
3. More than 10 years of treatment with metformin at time of screening
4. History of intolerance or allergy or other contraindications to any of the proposed study medications
5. Resides in the same household with another GRADE study participant
6. Current need for any specific glucose-lowering medications solely for other conditions, for example for polycystic ovary syndrome
7. Symptomatic hyperglycemia requiring immediate therapy during screening or run-in, in the judgment of the physician
8. A life-threatening event within 30 days prior to screening or currently planned major surgery
9. Any major cardiovascular event in previous year, including history of myocardial infarction, stroke, or vascular procedure such as coronary artery or peripheral bypass grafting, stent placements (peripheral or coronary) or angioplasty.
10. Plans for pregnancy during the course of the study for women of child-bearing potential
11. History of or planning bariatric surgery, including banding procedures or surgical gastric and/or intestinal bypass (if banding removed, may be considered eligible after 1 year).
12. History of congestive heart failure (NYHA 3 or greater)
13. History of pancreatitis
14. Any new diagnosis of cancer in the previous 5 years (other than non-melanoma skin cancer), or treatment for any cancer in the previous 5 years (other than non-melanoma skin cancer). Exceptions may be made, at the discretion of the local Principal Investigator and after review by the subcommittee overseeing protocol implementation, for cancers, such as some thyroid cancers, that have a benign clinical course and are not expected to interfere with conduct of the study.
15. Personal or family history of MEN-2 or family history of medullary thyroid cancer
16. Estimated GFR (eGFR) <30 ml/min/1.73 m^2 or end stage renal disease requiring renal replacement therapy
17. History of severe liver disease or acute hepatitis or ALT >3 times upper limit of normal
18. Current alcoholism or excessive alcohol intake
19. Previous organ transplant
20. Treatment with oral or systemic glucocorticoids (other than short-term treatment, for example for poison ivy) or disease likely to require periodic or regular glucocorticoid therapy (inhaled steroids and/or physiological replacement treatment are allowed, e.g. for Addison's disease)

21. Treatment with atypical antipsychotics known to be associated with a high risk of metabolic dysfunction
22. History of hemolytic anemia, chronic transfusion requirement, or other condition rendering HbA1c results unreliable as indicator of chronic glucose levels, or hematocrit <35 for males and <33 for females
23. Clinically or medically unstable with expected survival <1 year
24. Unwillingness to permit sites to contact the PCP to communicate information about the study and the participant's data
25. At the time of final run-in, no identified PCP to provide non-study care. (Note: in cases where a study MD serves as the participant's PCP, another study provider must assume GRADE management decisions for the participant during the study.)
26. Participation in another interventional clinical trial
27. Previous randomization in the GRADE study
28. In the opinion of the principal investigator (PI), any other factor, including language barrier, likely to limit compliance with the protocol

7.0 RECRUITMENT

Potential subjects will be recruited from participating clinical centers after protocol approval by local IRBs. Clinical centers will employ electronic databases, community-based advertising, social media, mailings, and other means of local recruitment. Based on the high frequency of newly diagnosed diabetic patients in cardiology clinics and primary care practices, we will emphasize screening and recruitment from these settings (41).

7.1 Screening

Screening will include reviewing medical histories from the volunteers (most will have had a diagnosis of diabetes based on clinical criteria), focusing on age, timing of the diagnosis of diabetes, and on medication use. The use of electronic medical records and other databases will be recommended to make preliminary screening as efficient as possible. At the initial screening visit, HbA1c, liver function tests (ALT), hematocrit, and serum creatinine/eGFR will be measured locally and pregnancy testing in women with reproductive potential performed (Tables 1 and 2). Screening for the presence of diabetes will be facilitated by using HbA1c values $\geq$6.5%. The screening histories will be recorded on study forms. Once eligibility has been confirmed, subjects will proceed to the run-in period to determine suitability for randomization.

7.2 Run-in

The run-in period will be used to determine eligibility and ability to adhere to the study protocol (Table 1). Subjects will adjust metformin IR or metformin XR doses to 2000 mg daily, as tolerated. Subjects who cannot tolerate metformin IR to achieve the protocol-recommended 2000 mg per day will have a trial of metformin XR. Subjects who cannot tolerate at least 1000 mg daily of immediate or extended release metformin by the time of final run-in will be excluded from further participation. Subjects who have been treated with >2000 mg per day will have their dose adjusted to 2000 mg. The targeted time from initial run-in to the final run-in visit will be 4 weeks for participants who are on 2000 mg of metformin per day at screening, and 6-8 weeks to allow equilibration of the HbA1c on the study metformin for those who require titration. The run-in period will also be used to familiarize the volunteers with the study and the study staff with the potential participants. In addition, the diabetes education program that will be provided to all participants will take place during the run-in period, before randomization. In some cases, such as temporary condition or blood test that resulted in an ineligible status, a repeat screening visit and/or blood test may be scheduled.

7.3 Randomization

Eligible study participants will be randomized at the baseline visit, and will be assigned a study randomization number to which a treatment group assignment has been made. Randomization will be stratified by site and conducted via a central web-based system. The participant, the clinical investigator, and clinical personnel will not be masked to the treatment assignment. Laboratories performing assays for this protocol will be masked to the treatment assignment and the identity of each participant whose biological material is to be studied.

Table 1: Run-in Schedule

Visit Type	Activities
Screen Visit	Screen/Run-in Consent<br>Confirm eligibility<br>Collect blood (local lab)<br>HbA1c*<br>ALT*<br>Hematocrit*<br>Serum creatinine/eGFR*<br>Urine pregnancy test (if applicable)*<br>Blood pressure<br>Weight
Initial Run-in Visit	Convert to study metformin and adjust dose (if < or >2000 mg daily)[+]<br>Teach titration protocol as needed<br>Demo of injection and finger stick or alternative site testing[‡]
Interim contact (phone and/or visit depending on titration schedule)	Ascertain tolerability of metformin and adherence to titration protocol
Final Run-in Visit<br>Length of run-in period will vary depending on metformin dose at screening	Assess adherence[+]<br>Standard diabetes education[$]<br>Collect blood samples (CBL)<br>HbA1c[±]<br>Serum creatinine/eGFR[±]<br>Consent (Clinical trial)[†]
Baseline Randomization Visit.<br>All should occur within approximately 5 to 45 days from blood sample collections at final run-in visit; and between approximately 36 to 98 days from the Screening Visit blood sample collections	Collect fasting blood/urine samples (CBL)<br>DNA[±]<br>Lipids[±]<br>Plasma glucose[±]<br>Urine albumin/creatinine[±]<br>Blood/urine and stool sample in subset of cohort for storage[±]<br>Oral Glucose Tolerance Test<br>Urine pregnancy test (kit from CBL)*<br>Questionnaires^<br>Waist/hip circumference^<br>Blood pressure, height, weight, ECG^<br>Neuropathy assessment^<br>Neurocognitive assessment^<br>Randomization to treatment<br>Dispense study medication

eGFR- Estimated Glomerular Filtration rate is calculated from serum creatinine.
CBL – Central Biochemistry Laboratory.
*Performed locally.
[±]Performed centrally.
[+]Study goal is 2000 mg per day, minimum 1000 mg per day for at least 8 weeks at final run-in.
[†]Consent for the clinical trial (phase 2) may be obtained at either final run-in or baseline visit (prior to randomization)
[‡] Can be done at any visit during run-in, preferably at visit 1. ^Can be administered at final run-in visit.
[$] May be conducted at Initial Run-In Visit or during Interim Run-In Visits

8.0 OUTCOME ASSESSMENTS

8.1 PROTOCOL OUTCOME MEASUREMENTS AND ASSESSMENTS

Table 2 lists the outcome measurements and assessments to be performed during the trial and the schedule of assessments for each. The study defined outcomes and the analytic plan are in Chapter 13. The measurements and assessments can be grouped as follows.

Metabolic

- HbA1c
- Fasting plasma glucose (FPG) values
- Hypoglycemia episodes
  - Symptomatic
    - Probable (relieved by food or glucose tablets)
    - Confirmed (with BG <70 mg/dl)
  - Severe (requires third party assistance)
    - Major (severe episodes that result in loss of consciousness and/or seizure)
    - Results in injury to the participant or others (e.g. motor vehicle accident in which the participant was the driver)
- Body weight, waist and hip circumferences, body mass index (BMI)
- Measurements derived from the fasting measures and OGTT to assess insulin resistance, beta-cell function, and other metabolic parameters

Cardiovascular

- Blood pressure, use of anti-hypertensive agents
- Lipid profiles, use of drugs to treat dyslipidemia
- Major adverse cardiovascular events (MACE) - cardiovascular death, nonfatal MI, nonfatal stroke
- Other cardiovascular events including unstable angina requiring hospitalization or revascularization
- Congestive heart failure requiring hospitalization
- ECG abnormalities, using the Minnesota ECG classification, including myocardial infarction, myocardial ischemia, left ventricular hypertrophy, arrhythmias, and conduction defects
- Assessment of cardiac autonomic function as measured by ECG

Microvascular

- Albumin:creatinine ratio
- Serum creatinine and estimated glomerular filtration rate (eGFR)
- Retinal photocoagulation for diabetic retinopathy and other ophthalmologic procedures by self-report (42)
- Peripheral neuropathy as assessed by modified MNSI, including 10 gm monofilament (<8/10 abnormal), vibration sensation threshold and presence of ankle reflexes

Adverse effects

- Pancreatitis

Side-effect profile (other than hypoglycemia or weight change)

- Gastrointestinal
- Other

Adherence-tolerability

- Adherence to study medications
- Tolerance of study medications

Health-economic

- Costs of therapy
- Quality of life (e.g. SF-36, QWB) (43)

Other

- Mortality
- Hospital admissions
- Cognitive function measured with battery of tests developed for the Diabetes Prevention Program Outcome Study (44)
- Diagnosis of cancer (by type, tracking all cancers except non-melanoma skin cancer)
- Frequency of taking other glucose-lowering drugs (that may have been prescribed outside of study), lipid lowering, blood pressure lowering medications and those targeting the renin-angiotension system.
- Treatment satisfaction

All subjects will be followed through study end for the ascertainment of the designated outcomes including microvascular outcomes and CVD and cerebrovascular events and effects of rescue therapy.

Electrocardiograms (ECG) will be done at baseline and at selected annual visits, depending on funding availability.

8.2 Other Metabolic Measures (for phenotyping and determination of mechanisms of success or failure of interventions)

Currently, the majority of subjects with type 2 diabetes are treated similarly, as if they all have the same metabolic abnormalities, despite substantial data that show that type 2 diabetes is a highly heterogeneous disease. There are limited data to guide the choice of therapeutic agents that are most likely to lower glycemia successfully in a specific individual or subgroup. Such information would allow tailoring of medication regimens to provide the most efficient therapies. In addition to studying demographic, anthropometric, and other clinical variables (e.g. blood pressure, and nondiabetic medications), we will examine other hypothesis-driven metabolic characteristics at baseline in all subjects and perform detailed follow-up phenotyping (potentially in subsets, depending on funding availability). The same measurements will provide valuable information regarding the mechanisms of action (crudely, effects on insulin resistance versus insulin release) of the individual combinations and, as they fail, the metabolic cause(s) of the failure.

Baseline and follow-up measurements of phenotypic variables (demographic, physiologic, and genetic) will facilitate the study of patient factors that mediate responsiveness to different therapies (Table 2). They may also provide insights regarding the mechanisms of individual drug combination success and of drug failure over time. Demographic, clinical and historical data may include age, sex, race, insurance status, years of education, occupation, marital status, consumption of alcohol, tobacco, and other drugs known to affect glycemia or

cardiovascular disease risk, overweight or obesity, cardiovascular disease, diet and exercise behaviors, allergies, and other medications, and family history of diabetes.

Stool specimens will be saved in a subset of GRADE participants at baseline and during follow-up to allow examination of the potential effects of an individual's microbiome on response to drug therapy and how the randomized therapies used affect the gut microbiome and other metabolic parameters.

Table 2: Schedule of Measurements and Assessments

Measurements and Assessments	Screen	Final run-in	Base-line	1 Y	2 Y	3 Y	4 Y	5 Y	6 Y	7 Y
HbA1c¶	L	C		Q/C	Q/C	Q/ C	Q/C	Q/C	Q/C	Q/C
Oral Glucose Tolerance Test (OGTT)±			C	C		C		C		
DNA			C							
Fasting lipids			C	C	C	C	C	C	C	C
Liver function tests (ALT)	L									
Serum creatinine/eGFR (safety labs)	L	C		C	C	C	C	C	C	C
Albumin:creatinine (urine)			C	C/S	C/S	C/S	C/S	C/S	C/S	C/S
Blood and urine samples for storage#			C	C	C	C	C	C	C	C
Stool samples in subset of participants for storage#			C/S§							
Urine pregnancy@	L		L							
Hematocrit	L									
Vitamin B12				C¥			C			
History (events-medications)	L		L	L/Q	L/Q	L/Q	L/Q	L/Q	L/Q	L/Q
Physical Assessment (BP, Weight)	L		L*	L/Q	L/Q	L/Q	L/Q	L/Q	L/Q	L/Q
Height			L*				L			
Waist and hip circumference			L*		L		L		L	
Peripheral neuropathy			L*	L	L	L	L	L	L	L
ECG (read centrally) ‡‡			L*/C		L/C‡‡		L/C‡‡		L/C‡‡	
Study drug adherence		L	L	L/Q	L/Q	L/Q	L/Q	L/Q	L/Q	L/Q
Cognitive Battery			L*				L		L	
QOL-SF-36, QWB, Treatment Satisfaction+			L*	L	L	L	L	L	L	L

C=Performed centrally, L=Performed locally, Q=quarterly, S= semi-annually
¶Quarterly. Needs confirmation at next (3 month) visit if ≥7% (in 3-6 weeks if >9%). Remote capillary collection procedures may be used in circumstances when the participant is unable to come to the clinical site.
#Biological samples saved as aliquots for substudies/ancillary studies.
§Stool samples and microbiome related data will be collected on a subset of participants at baseline and 6 months, with additional samples, funding permitting.
±OGTT will include insulin, C-peptide, and glucose measures at 0 (fasting), 2 hours, and other selected times; however, insulin is not measured in insulin-treated subjects

@As indicated. +DTSQ performed at baseline, 6 months, and 1 year.
*May be completed at the Final Run-in visit
¥Will be tested at intervals during the study depending on the participant's randomization date.
‡‡ Conducted at baseline and selected annual visits during the study pending availability of funding.

An Oral Glucose Tolerance Test (OGTT) will be performed at baseline and at the year 1, 3 and 5 annual visits. During each OGTT, timed collections of serum and plasma will be used to measure glucose, insulin and C-peptide. From these assessments, a number of different outcome measurements can be obtained with the goal of assessing the differential metabolic effects of each drug combination on beta-cell function and insulin resistance over time:

- HOMA measures of beta-cell function and insulin sensitivity (45)
- Oral glucose disposal efficiency calculated as the incremental area under the curve (AUC) glucose above fasting. This provides a measure of overall glucose tolerance as the interventions may affect fasting and glucose excursions differentially
- Insulinogenic index calculated over the first 30 minutes after glucose ingestion ($\Delta insulin_{30-0}/\Delta glucose_{30-0}$)
- Insulin sensitivity index as 1/fasting insulin
- Oral disposition index (DIo) as a measure of beta-cell function. This is calculated as the product of the insulinogenic index and insulin sensitivity index (46).
- Mathematical model determinations of insulin sensitivity and beta-cell function using glucose, insulin and C-peptide (e.g.(47, 48)
- Hepatic insulin clearance based on ratios of C-peptide and insulin
- Glucagon suppression may also be determined, resources permitting, as the incremental/decremental glucagon AUC from the fasting value as a measure of alpha-cell function. Several of the study drugs (GLP-1 receptor agonists and DPP-4 inhibitors) would be expected to affect this measure.

9.0 STANDARD DIABETES EDUCATION

All participants will be provided standard diabetes education during the run-in period and annually thereafter. Standardized materials will be available to study coordinators that will include information on the pathophysiology of diabetes, prevention of complications, reduction of CVD risk factors, diet/nutrition, exercise goals, and self-care such as foot care and medication taking. The importance of eating a healthy diet, losing weight if overweight or obese, and being physically active are stressed. All individuals who smoke will be encouraged to stop smoking and provided with self-help materials and/or referral to local programs, as appropriate. The initial standard diabetes education during run-in will include instructions regarding self-monitoring of blood glucose (SMBG), management of hypoglycemia and the importance of medication adherence. For subjects who are at risk for hypoglycemia (those randomly assigned to sulfonylurea or insulin therapy), SMBG will be reinforced after randomization. Sites will have the option to provide education in group or individual sessions and to prioritize the order and frequency of content delivery.

10.0 ADVERSE EVENT REPORTING

10.1 Serious Adverse Event Reporting

Serious adverse events include events resulting in death or which are life threatening, or result in hospitalizations (or emergency room visits) that last ≥24 hours, or prolongation of a hospitalization, or persistent or significant disability/incapacity or a congenital anomaly/birth defect. The occurrence of hyperglycemic emergencies (diabetic ketoacidosis or hyperosmolar hyperglycemic syndrome), major hypoglycemia (i.e. severe hypoglycemia resulting in loss of consciousness and/or seizure), severe hypoglycemia that results in injury to the participant or others (e.g. a motor vehicle accident in which the participant was the driver), or lactic acidosis or major cardiovascular events (non-fatal stroke or myocardial infarction) will also be considered serious adverse events. All of these events will be reported by participants at any time following the initial run-in visit, and will specifically be collected at quarterly visits during study follow-up.

10.2 Other Adverse Events

Other adverse events, including potential side effects of medications, will be collected in a standardized fashion using a structured questionnaire that includes side-effects that have been noted at a >5% rate in the clinical trials of each drug that led to their approval to be marketed.

11.0 OTHER MEASUREMENTS AND ANCILLARY STUDIES

The scientific and clinical importance of the trial will be amplified through the development and performance of potentially more intrusive and complicated procedures in selected samples of the cohort ("substudies") and through ancillary studies. In considering the selection of these substudies, the study group will take into account the potential impact on the participants, excluding procedures that might adversely affect continued participation in the study. In addition, procedures that could be performed at the majority of centers will be preferred.

Ancillary studies (usually proposed by Research Group members) will require review and approval by the study to ensure that they will not interfere with the successful conduct of the main study. Those studies that are approved will require funding independent of the main funding of the trial, for example through the NIDDK ancillary studies funding mechanism or other R01 support. These studies may take advantage of the saved biosamples or may request new samples/measurements.

12.0 STUDY OUTCOMES

12.1 Primary Outcome

The primary outcome is the time to primary metabolic failure of the randomly assigned treatment, defined as the time to an initial HbA1c ≥7%, subsequently confirmed at the next quarterly visit, while being treated at maximum tolerable doses of both metformin and the second randomly assigned medication (intention-to-treat). If the second (confirmatory) HbA1c is <7%, then the primary outcome is not reached.

If the initially observed HbA1c is >9% while being treated at maximum tolerable doses of the assigned regimen, then the confirmation value will be performed within 3 to 6 weeks. If the

initial HbA1c and confirmation value 3 to 6 weeks later are both >9%, the primary outcome and secondary outcome will have been reached. If the initial HbA1c is >9% and the confirmation value 3 to 6 weeks later is ≤9%, the participant will resume his usual schedule of quarterly HbA1c monitoring. If the HbA1c at the next quarterly visit is >7% while receiving the maximally tolerated doses of the assigned regimen, then the primary outcome will have been reached.

The primary outcome can only be reached after a minimum of 6 months of therapy, unless the HbA1c at 3 months is >9% and is higher for the confirmation HbA1c 3-6 weeks later, in which case the primary and secondary outcomes will have been met at 3 months. All results will be measured in the study central laboratory.

Diabetes medications are to be titrated to maximally tolerated doses. The maximally tolerated dose is defined as the highest dose at which the participant is without unacceptable side effects and which the participant is willing to take. Doses may be decreased in response to side effects or increased, as tolerable, to achieve the goal glycemic target at any time during the study.

An adjudication committee, masked to intervention, will determine if subjects have reached the primary outcome if a second HbA1c value is not available. Information on laboratory results obtained outside of the study, or diabetes-related or other medications started by non-study care providers that might have interfered with the primary outcome will be sought.

12.2 Other Metabolic Outcomes

- Proportion of subjects among those randomized to each treatment that has reached the primary metabolic outcome over time
- Time to secondary metabolic failure (HbA1c >7.5%, confirmed) after having reached the primary outcome, while receiving the maximally tolerated dose of the assigned regimen. The primary and secondary metabolic outcomes may be reached simultaneously if the initial value and the confirmation are both >7.5%.
- Proportion of subjects among those randomized to each treatment that has reached secondary metabolic failure over time.
- Time to the need for intensive insulin therapy (defined as basal plus rapid-acting insulin), while being treated at maximum tolerable doses of the assigned regimen.
- Proportion of subjects among those randomized to each treatment group that has initiated intensive insulin therapy over time.

12.3 Health-Economic Evaluation

A “within trial” economic evaluation will be integrated into the clinical trial to assess the cost-effectiveness of each therapy over an average period of at least 4.8 years of treatment and follow-up. We will prospectively collect data on the volume of resources used in each group, including additional calls and visits, as part of the study case report forms, and detailed resource utilization, and cost data will be collected midway during the study from a sample of participating sites. Additional costs will be obtained from national sources. In addition, change in quality of life using established instruments (e.g. QWB (43)) will be administered that will allow patient self assessments to be employed in the calculation of quality adjusted life years. Such health economic analyses have been integrated successfully into previous trials (49).

12.4 Secondary Composite Outcomes

In addition to the primary and secondary clinical outcomes based on failure to maintain metabolic control, comparative effectiveness analyses incorporate other characteristics of the interventions. A simple secondary outcome that reflects both durability of glycemic control and tolerability to the assigned medications is the simple proportion of patients originally randomized to a treatment group who are still able to maintain Hb1c < 7% on the originally assigned regimen after 4 or more years of follow-up.

However, such a simple index will not capture all of the facets of response to therapy. For example, if glycemic control over time proves to be similar among several or all treatment groups, an important objective of this comparative effectiveness trial will be to determine how each treatment regimen affects other domains of diabetes care, overall health, and cost. Unfortunately, there is no currently accepted standard composite outcome to reflect all facets of diabetes care.

Although it would be ideal to use hard outcomes, such as cardiovascular disease events and mortality, for every diabetes trial, the sample size needed to accrue sufficient numbers of such outcomes to provide adequate power to detect treatment differences, especially early in the course of diabetes, is prohibitively large. Several risk factor "engines" for microvascular and cardiovascular outcomes have been developed, predominantly from epidemiologic data (50, 51). Although some of these have been used as secondary outcomes in clinical trials, it is not clear how well they would perform in the current study, i.e., whether they would be differentially sensitive to the interventions. Moreover, the outcomes targeted by these risk factor engines would only include a subset of the outcomes of interest and would not capture tolerability, acceptability and the relative safety of regimens.

To avoid imprecision and misleading conclusions, several groups of authors have suggested that composite outcomes include "components that are similar in importance to patients, that occur with similar frequency, and that are affected to a similar degree by the intervention" (52, 53). There are several overlapping domains of diabetes outcomes meeting these criteria that will serve as important secondary outcomes of the trial. For each domain, a multivariate analysis will be performed to assess whether there are differences among groups in the set of characteristics assessed in that domain (see the statistical analysis section to follow). Each of the component events, e.g., moderate or severe hypoglycemia, is defined in a subsequent section. Since the secondary outcomes are heterogeneous and cannot be incorporated into a single metric, they must be considered separately. Choosing a hierarchy of clinical importance of these secondary outcomes is obviously arbitrary and depends on perspective (e.g. societal vs. patient). We have listed below the secondary outcomes in their order of clinical importance (1-7), recognizing the arbitrary nature of this ordering.

1. ***Direct effects of glucose-lowering agents***. Glycemic control measured as the mean HbA1c, the mean body weight and the rate per year of moderate (or more severe) hypoglycemia since randomization over time, such as up to 4 years of follow-up. We will compute a mean HbA1c value and mean weight for each participant, and an overall rate of hypoglycemia events, i.e., one value each per subject. The analysis will then assess whether one treatment has a greater beneficial effect than another on all three outcomes simultaneously. For the HbA1c and weight, we will also analyze the actual level of HbA1c and weight adjusted for the baseline level. Such analyses will also be conducted up to the time of primary outcome or end of study

   An additional outcome will be the time to either an episode of severe hypoglycemia or the time of the primary outcome, whichever occurs first. The analysis would then

describe the proportion of participants that both remain in good control without having experienced any hypoglycemia over time. A similar outcome could include the time to weight gain of 5% of body weight from baseline.

2. ***Mechanisms***. Physiologic or mechanistic variables will be assessed as predictors of time to glycemic deterioration by medication class and across classes. Exploring these variables may provide information to help clinicians in selecting the medication that will work best for that individual patient. This will promote a more detailed understanding of the mechanisms by which the drug classes do or do not prolong the time to such glycemic deteriorations, and to define different metabolic phenotypes with varying risk of such deterioration. In addition, we will assess whether common clinical, anthropomorphic and demographic characteristics such as fasting and stimulated glucose and insulin levels, BMI, waist circumference, age and sex can be used in combination to predict time to glycemic deterioration by medication class and across medication classes, so as to allow risk classification of patients with recent-onset diabetes in clinical settings.

3. ***Effects on traditional and other cardiovascular risk factors and macrovascular complications.*** The incidence of hypertension, hyperlipidemia and microalbuminuria based on accepted clinical definitions (defined by dichotomous values and taking into account medication use) and of major cardiovascular events will be analyzed. While the study is not designed to provide a definitive assessment of relative cardiovascular risks/benefits of the different treatments, the study will provide minimally adequate power to detect large differences in risk between treatment groups.

4. ***Specific adverse effects of drugs***. Incidence of gastrointestinal symptoms (including nausea, vomiting, diarrhea), and pancreatitis summarized as an overall rate per year for each subject.

5. ***Adherence, tolerability, and acceptability of treatment.*** Adherence is measured as the proportion of participants who take their assigned medications. Tolerability and acceptability will be assessed by periodically administered questionnaires.

6. ***Global and diabetes-specific quality of life***. Diabetes quality of life and general assessments (e.g. QWB and SF-36) will be measured.

7. ***Patient-oriented outcome assessments***. Participants will be asked periodically to provide their own assessment of the utility of and overall satisfaction with the assigned regimen (e.g. DTSQ).

12.5 Microvascular and Cardiovascular Outcomes

Although desirable, funding considerations preclude a comprehensive assessment of long-term micro- and cardio-vascular outcomes as part of the main study. However, we will assess albumin:creatinine ratio (ACR) centrally every 6 months throughout the study as an objective, affordable, practical, and clinically important outcome. The measurements will be obtained until end of study, including visits after a patient has reached the primary metabolic outcome. The frequent measurement of microalbuminuria will allow determination of the time of onset of microalbuminuria as the visit at which a patient has an ACR value >30 mg/g among

patients who enter the study with a baseline ACR ≤30 mg/g and the time to the development of "confirmed" microalbuminuria with two consecutive measurements >30 mg/g.

We will also assess retinopathic outcomes (e.g. photocoagulation therapy) by patient self-report. Neuropathy will be assessed using the 10-gram monofilament examination for the presence of peripheral neuropathy.

Cardiovascular outcomes will include major adverse cardiovascular events (MACE), including fatal and non-fatal myocardial infarctions and stroke. These will be reported as they occur and/or collected as part of the structured medical history obtained at each visit.

ECGs will be performed to establish baseline cardiovascular status and to detect development of new findings at the follow up visits. The Minnesota ECG classification will be used as the basis for detection of ECG abnormalities and will provide several measurements that are known to reflect underlying cardiac pathology. In addition, ECG will provide measures to assess cardiac autonomic function.

GRADE investigators may also submit applications for additional funding to perform further assessments of microvascular disease (e.g. fundus photography) or cardiovascular disease (e.g. cardiac CT).

12.6 Other Outcomes (Table 2 lists the measurement frequency for outcomes)

In general, the individual components of the aggregate (secondary) outcomes will be analyzed as other outcomes.

Metabolic (other than those listed in 12.2)

- Overall mean HbA1c values over the duration of the trial
- Mean change in HbA1c from baseline to year 3
- Overall mean fasting plasma glucose (FPG) values over time
- Hypoglycemia rates per patient year (or 100 years)
  - Symptomatic
    - Probable (relieved by food or glucose tablets)
    - Confirmed (with BG <70 mg/dl)
  - Severe (requires third party assistance)
    - Major (severe episodes that result in loss of consciousness and/or seizure)
    - Results in injury to the participant or others (e.g. motor vehicle accident in which the participant was the driver)
- Mean change in body weight, waist and hip circumferences, body mass index (BMI) over time
- Proportion of subjects who develop obesity (BMI $\geq$30 kg/m^2) or greater levels of obesity (e.g. $\geq$35 kg/m^2) of those without the condition at baseline
- Measurements derived from the fasting measures and OGTT to assess insulin resistance, beta-cell function, and other metabolic parameters such as alpha-cell function, funding permitting
- Effect of rescue therapy on mean HbA1c and other metabolic outcomes above

Cardiovascular

- Change in blood pressure, use of blood pressure-lowering agents, over time

- Incidence and prevalence of hypertension defined as blood pressure of ≥140 mmHg systolic or ≥90 mmHg diastolic OR the use of blood pressure-lowering medications for control of blood pressure
- Incidence of emergent hypertension among those who had levels <140/90 and were free of blood pressure-lowering medication use at baseline
- Change in lipid profiles, use of drugs to treat dyslipidemia
- Incidence and prevalence of hyperlipidemia defined as LDL-cholesterol levels ≥100 mg/dl or the use of lipid-lowering medications
- Incidence of emergent hyperlipidemia among those who had LDL levels <100 mg/dl and were free of lipid-lowering medication use at baseline
- Incidence of major adverse cardiovascular events (MACE - cardiovascular death, nonfatal MI, nonfatal stroke)
- Incidence of ECG-detected abnormalities, including silent MI, myocardial ischemia, left ventricular hypertrophy, arrhythmias, and conduction defects
- Incidence of cardiac autonomic dysfunction
- Incidence of other cardiovascular events including unstable angina requiring hospitalization or revascularization
- Incidence of congestive heart failure requiring hospitalization
- CVD risk calculated using the UKPDS, Framingham or other cardiovascular risk engine (50, 51)

Microvascular
- Change in albumin:creatinine ratio over time
- Incidence of microalbuminuria (>30 mg/g albumin:creatinine) among subjects who had levels <30 at baseline
- Incidence of “confirmed” microalbuminuria (microalbuminuria at two consecutive measurements) over time
- Incidence of macroalbuminuria (>300 mg/g albumin:creatinine) among subjects who had levels <300 at baseline
- Change in measurement of renal function including estimated glomerular filtration rate over time
- Incidence of retinal photocoagulation for diabetic retinopathy and other ophthalmologic procedures by self-report. Self-report of laser treatment is 96-99% accurate (42)
- Incidence of peripheral neuropathy based on an abnormal modified MNSI (including 10 gm monofilament test, <8/10 constitutes an abnormal result), ankle reflexes and vibration sensation (54)

Adverse effects – Incidence of
- Pancreatitis
- Pancreatic and medullary thyroid cancer
- Cancer (by type, tracking all cancers except non-melanoma skin cancer)

Side-effect profile (other than hypoglycemia or weight change)
- Gastrointestinal
- Other

Adherence-tolerability
- Adherence to study medications
- Tolerance of study medications
- Treatment satisfaction and quality of life

Health-economic

- Cost and cost-effectiveness
- Change in quality of life (e.g. SF-36, QWB) (43)

Other

- All-cause mortality
- Any hospital admission
- Change in cognitive function measured with battery of tests developed for DPPOS (44)
- Diagnosis of cancer (by type, tracking all cancers except non-melanoma skin cancer)
- Frequency of taking other (non-study) glucose-lowering medications

Any events that are adjudicated will be masked to treatment assignment.

13.0 STATISTICAL ANALYSIS PLANS

A separate document, the *Statistical Analysis Plan*, provides a detailed and comprehensive description of the statistical approach to address the various study objectives and the statistical considerations for analysis of the study data. This *Statistical Analysis Plan* supersedes the following section of the protocol that provided an initial descriptions of the analysis plans. The *Statistical Analysis Plan* will be locked (fixed) prior to the final closure of the study data base after the completion of follow-up of the study cohort.

13.1 General Analysis Strategies

For each outcome, analyses will be conducted to assess differences among the four drug class combinations with metformin. All analyses will be conducted under the intention-to-treat principle using the treatment as assigned to each subject, and using all available data from all subjects.

Additional analyses of study data will be conducted to address the primary and secondary objectives of the trial, other stated objectives, and other interrelationships among elements of study data of interest to the investigators and of relevance to the objectives of the study.

13.2 Primary Outcome

The primary outcome is the time to the observation of an HbA1c value $\geq$7%, subsequently confirmed, while subjects are treated with the maximally tolerated doses of both metformin (up to 2000 mg per day) and the randomly assigned medication. Since the HbA1c is measured quarterly, the event time will be the discrete quarterly follow-up visit number at which the test was conducted that exceeded the 7% outcome and was subsequently confirmed.

The cumulative incidence of the primary outcome within each treatment group will be estimated using a modified discrete time Kaplan-Meier estimate, allowing for periodic outcome assessments (55). Differences between groups will be tested, and relative risk estimates obtained, from a Cox proportional hazards model for discrete time observations, adjusted for the baseline HbA1c, with a 4 category class covariate to represent the 4 drug class groups (55).

A single overall omnibus test at the 0.05 significance level will be conducted comparing the 4 drug combination groups.

Significance tests and relative risk (hazard ratio) estimates for each of the 6 pairwise drug group comparisons will be obtained as contrasts among model coefficients from the overall Cox model. P-values from the pair-wise comparisons among the 4 drug treatments will be adjusted using the Holm closed sequential multiple testing procedure (56) that requires that the correction of 0.05/6 only be applied to the most significant of the 6 pair-wise tests, larger values for other tests.

If tests of the proportional hazards assumption do not apply, inferences (confidence intervals and p-values) will be obtained using the robust information sandwich estimates of standard errors (55).

13.3 Other Outcomes

The above analysis strategy will also be applied to other discrete time-to-event outcomes, such as the time to secondary metabolic failure or the observation of microalbuminuria based on a 6-monthly albumin:creatinine ratio (ACR). In analyses of emergent events, such as microalbuminuria, subjects with the outcome event present at baseline are removed since they are not at risk of experiencing the event during follow-up. In addition, for a single outcome, such as microalbuminuria, the model will adjust for the baseline ACR value rather than adjusting for HbA1c.

For time-to event outcomes measured nearly continuously, such as the number of days to a cardiovascular event, the above strategy will be employed using the corresponding methods for continuous time observations. For such analysis of cardiovascular outcomes, the PH model will also adjust for baseline age as well as HbA1c.

For longitudinal analyses of binary outcomes over time, such as the proportion of subjects (prevalence) at each visit who are still maintaining HbA1c <7% while receiving the originally assigned therapy, the odds will be compared between groups using a repeated measures logistic model fit through generalized estimating equations with a robust estimate of the covariance structure (57).

For longitudinal analyses of quantitative secondary outcomes over time (such as HbA1c) a longitudinal normal errors repeated measures model will be employed that adjusts for the baseline value and visit time, as above, for the estimation of group mean levels over time (58). A logarithmic or other transformation will be employed, as appropriate to satisfy the model assumptions, or alternately a model will be fit using generalized estimation equations (57) and inferences based on the robust information sandwich variance estimates.

For longitudinal assessments of the rate of change of an outcome over time, such as the slope of the decline in estimated GFR, a random effects (random coefficient) model will be used to estimate the mean slope within each treatment group, allowing for random variation of slopes among subjects (58).

For the comparison of rates of events (e.g. hypoglycemia), Poisson regression models will be employed using the robust information sandwich variance estimates (55).

13.4 Composite Outcomes

For a multivariate composite outcome, such as HbA1c, weight and hypoglycemia, a single multivariate test will be conducted aimed at detecting improvement in all components simultaneously for one drug treatment versus another. Such tests are referred to as a multivariate one-sided (or one-directional) test, or a test of stochastic ordering. One simple such test is that of O'Brien (59) that is based on each subject's mean of the rank scores for each component. This is suitable for an analysis of multiple quantitative (or ordinal) components at a single point in time. Alternatively the Wei-Lachin test of stochastic ordering could be used to conduct a one-directional test of multiple components using a different analysis for each component, such as a test of difference between means for one component, test for proportions or event-times for another, or a test of incidence rates for another (60). For the composite above, the difference in the mean HbA1c and mean weight at 4 years of follow-up, and the rate of hypoglycemia per 100 patient-years over the 4 years of follow-up, could be assessed jointly. An analysis with adjustment for baseline covariates will be conducted (60).

In addition the incidence or prevalence of a composite outcome will be assessed using a single combined outcome, such as the prevalence of subjects at 4 years who are still able to maintain an HbA1c <7% without having experienced any hypoglycemia or gained any weight. A longitudinal analysis will be conducted of the proportions meeting this criterion at each visit over time, and a "survival" analysis will also be conducted based on the time to failure to maintain this composite outcome (i.e. the time to either the primary outcome or hypoglycemia or weight gain).

Proportional hazards and parametric regression models will be used to assess the ability of multiple variables simultaneously to predict the time to primary or to secondary failure. Models will be developed mindful of the biases that can be introduced by data dependent model selection. Models will be validated by cross validation and the risk estimates will be calibrated for accuracy.

13.5 Interim Analyses

A data and safety monitoring board (DSMB) will be appointed by the NIDDK and charged with monitoring trial conduct and performance, and evaluating the safety of participants. Since this is a study of FDA-approved medications that are administered according to the FDA-approved labeling, with long-term scientific objectives, there is no need for the DSMB to consider early termination based on the appearance of a beneficial effect of treatment (effectiveness), or the absence of a beneficial effect (futility).

13.6 Subgroup and Stratified Analyses

Analyses will also be conducted assessing the differences in study outcomes within segments of the study cohort defined from characteristics assessed at baseline, such as by gender. For each stratification factor (e.g. gender), the treatment groups will be compared separately within each stratum or subgroup (e.g. males and females) and then a test of homogeneity between strata (no stratum by group interaction) will be tested. Initially the within stratum and between strata tests will be conducted using a multivariate test of the equality (and homogeneity) of the differences among the 4 treatment groups simultaneously. If any heterogeneity is detected, then additional tests will be conducted separately for each of the 6 pairwise drug group comparisons. Such tests can be conducted using an appropriate regression model for each outcome, such as a Cox proportional hazards model for the time to

primary metabolic failure. For strata defined from a quantitative variable (e.g. age), an additional test of interaction will be conducted using the quantitative covariate rather than simply the discrete strata.

The baseline factors to be considered include race/ethnicity, gender, age, diabetes duration, weight, BMI, HbA1c, and measures of insulin sensitivity, insulin secretion and the glucose disposal index, all measured at baseline or prior to randomization.

Age will specifically be stratified as <45, 45-59 and ≥60 years whereas other quantitative covariates will be stratified by tertiles.

14.0 SAMPLE SIZE AND POWER

Addendum 08/24/2016.

Owing to a 6 month delay in recruitment from that originally planned, the period of recruitment was extended by 6 months to 3.5 years with the estimate that 4800 or more participants would be enrolled. To preserve study power the total maximum follow-up period was likewise extended to 7.5 years. Calculations using the original design assumptions (hazard rates, etc.) show that the power of the study is preserved with the analysis of the primary outcome providing the same level of power (0.90) as in the original protocol. Likewise the power of other secondary outcomes is also largely preserved.

Thus, the original power calculations have been retained as in the text of the original protocol. They were based on 5000 subjects recruited over 3 years with 7 years of follow-up.

Sample size and power were assessed for the analysis of time-to-event outcomes using the method of Lachin and Foulkes (61) for the comparison of two groups, adjusting for 6 pair-wise comparisons in the analyses of the 4 drug groups.

The clinical centers have been recruited to enroll up to 5000 participants within approximately 3 years and to follow them for up to 7 years (under the original design). With continued follow-up of all subjects to study end, this would provide a minimum of about 4 and a maximum of 7 years of follow-up. With a constant rate of recruitment (linear) and no losses-to-follow-up, the mean follow-up period would be approximately 5 years. However, to be conservative, sample size and power for the primary analysis were computed assuming a lag in recruitment using the Lachin-Foulkes model (61) in which we assume that 40% of subjects are recruited in the first half of the 3-year recruitment period, 60% in the second half. We also assumed that 4% will be lost to follow-up before reaching the primary outcome. With these assumptions, the average follow-up time prior to the end of the study or loss-to-follow-up would be 4.8 years with 15% of subjects lost-to-follow-up. This loss-to-follow-up rate is considered realistic and sample size calculations were conducted using this value.

Under the same assumptions employed to assess power in the next section, we estimate that the average subject will either reach the primary metabolic outcome (≥7%) or end of study or be lost-to-follow-up after an average of 3.8 years in the study. Subjects reaching the initial HbA1c ≥7% will then be continued on the assigned medications for an additional three

months while the initial elevation is confirmed. After reaching this primary metabolic outcome, subjects will continue treatment on the original assigned regimens until a secondary metabolic outcome of HbA1c >7.5% (later confirmed) is also reached. If subjects take on average an additional 6 months to reach an HbA1c >7.5%, with another 3 months for confirmation, we estimate that the average subject will then initiate rescue therapy (basal insulin initiated for subjects assigned originally to medications other than insulin and intensive insulin started for subjects initially assigned to basal insulin) 4.8 years after randomization into the study (3.8 years average to primary outcome + 3 months confirmation + 6 months to secondary metabolic outcome + 3 months confirmation). On average, therefore, the mean period of treatment and follow-up (randomization to the study end date) will be 4.8 years. Thus, the period of rescue therapy, on average, is expected to be brief, although some subjects may have many years of such therapy. We estimate that 167 participants will reach the primary metabolic outcome within the first year of follow-up. If those subjects take another 9 months to reach the secondary metabolic outcome, including its confirmation, then those subjects will initiate rescue therapy on average at 21 months in the study.

14.1 Primary Outcome

The cumulative incidence of the primary outcome (failure to maintain an HbA1c <7% using two medications at maximum tolerated dose and confirmed) will be compared between groups using a Mantel-logrank test under a proportional hazards model (see Section 13.2). There are no preliminary data on the incidence rate with which subjects on combination therapy will reach the primary outcome of failure to maintain adequate glycemic control. Moreover, the vast majority of studies examining metabolic effects of combination therapy are 6-12 months in duration. However, there are some preliminary data from studies of monotherapy.

The ADOPT study compared the durability of glucose control using initial monotherapy with rosiglitazone versus metformin versus glyburide (19). Among those randomly assigned to metformin, based on the mean changes in HbA1c over time observed in ADOPT, we estimate that 50% of subjects would reach an HbA1c >7% by 3 to 4 years, corresponding to an annual hazard rate of 0.17 to 0.23. In the UKPDS, among those assigned to receive metformin, 56% had an HbA1c >7% at 3 years of follow-up, corresponding to a hazard rate of 0.274 (10). We expect that the introduction of a second medication as an early combination therapy will reduce this hazard. Thus, the sample size computations were conducted conservatively using a hazard rate of 0.0875, or approximately one-half of ADOPT's 0.173 hazard rate.

For an individual pairwise comparison among any two of the four drug groups, it is desirable to have 90% power to detect a 25% difference in risk (hazard rate) between groups, or a hazard ratio of 0.75. With a hazard rate for the primary outcome of 0.0875/y in the group with the higher incidence, a loss-to-follow-up hazard rate of 0.04 per year, a significance level of 0.00833 (0.05/6) adjusting for 6 pairwise tests, and assuming lagged recruitment over approximately 3 years with a total study duration of 7 years of follow-up from the first randomization, a sample size of 1242 per group would provide 90% power to detect a 25% risk difference. Of these, 416 subjects from the group with the higher hazard rate (0.0875/y) would reach the primary outcome compared to 329 subjects in the group with the 25% reduced risk. This corresponds to a total sample size of 4968, rounded to 5000 patients (1250 per treatment arm) with a total of 1663 reaching the primary outcome if all groups have the same risk (0.0875/y). If one group has a 25% risk reduction compared to the others, then we project 1576 subjects reaching this outcome; 1489 if 2 groups are likewise effective, and 1402 if 3 groups are effective.

However, the power of the study depends on other factors including the rate and period of recruitment, the hazard rate for losses to follow-up, the total hazard rate for the primary outcome and the total duration of treatment and follow-up. Based on the observed values of these factors (including the aggregate primary outcome hazard rate masked to treatment assignment), the period of recruitment and or total of treatment and follow-up (not to exceed 7 years) may be adjusted to optimize the power of the study.

14.2 Secondary Outcomes – Microalbuminuria and Clinical Cardiovascular Disease

The cumulative incidence of onset of microalbuminuria will also be compared among groups using a Mantel-logrank test under a proportional hazards model. From other studies, the hazard rate of onset of microalbuminuria is projected to be about 0.04 per year in whichever group has a higher event rate (62). For the 4-way comparison among the 4800-5000 subjects, the study would have 88% power with a hazard rate of 0.04/year, 92% with 0.045/year, to detect a 33% difference in risk for microalbuminuria between any pair of groups.

In the ADOPT study (19), the incidence of MACE was 0.76% per year and of MACE plus congestive heart failure was 1.14% per year. Assuming a more conservative incidence rate of 1% per year and the other assumptions above, GRADE will provide 80% power to detect a 50% difference in the risk of CVD between any pair of drug groups, adjusted for 6 pairwise comparisons. The study also has 80% power to detect a 42% difference in risk in an analysis of each drug group compared to all other drug groups combined, adjusted for 4 comparisons.

14.3 Subgroup Analyses

Assume that in the overall study one drug group has a hazard ratio for the primary outcome of 0.75 versus the other three drug groups. For a test of homogeneity of the 4-way drug group difference within two equal sized strata (subgroups) of 2500 subjects each, the study will provide 94% power to detect a pattern of drug group differences where the hazard ratio is 25% greater (HR = 0.938) within one stratum and 25% less (0.563) in the other. For the case of three strata with 1667 subjects each, the study provides 69% power to detect heterogeneity of hazard ratios of 0.563, 0.75, and 0.938.

15.0 POLICIES, ETHICAL CONSIDERATIONS, AND COMPLIANCE

15.1 IRB Approval

This study will be conducted in compliance with the protocol and all applicable regulatory requirements. Prior to study initiation, the protocol and the informed consent documents will be reviewed and approved by the Institutional Review Board (IRB) or an appropriate Independent Ethics Committee (IEC) at each participating clinical site.

15.2 Protocol Changes

Any amendments to the protocol or consent materials must be approved by an absolute two-thirds majority of the Steering Committee, and subsequently by the local IRBs before implementation at a site.

15.3 Participating Clinical Sites

Participating clinical sites must have a Federal-wide Assurance (FWA) with the Office for Human Research Protections (OHRP), since they are actively engaged in research and provide informed consent. The protocol and consent forms will be approved by Institutional Review Boards at each of the participating clinical sites. HIPAA regulations will be followed by each participating institution in accordance with each institution's requirements.

Each investigator is required to keep accurate records to ensure the conduct of the study is fully documented. The investigator is required to ensure that all case report forms are accurately and legibly completed for every participant entered in the trial.

The clinical sites participating in this study will maintain the highest degree of confidentiality permitted for the clinical and research information obtained from participants in this study. Medical and research records should be maintained at each site in the strictest confidence. However, as a part of the quality assurance and legal responsibilities of an investigation, the clinical site must permit authorized representatives of the sponsor(s) and regulatory agencies to examine (and, when required by applicable law, to copy) records for the purposes of quality assurance reviews, audits and evaluation of the study safety and progress. Unless required by the laws pertaining to copying of records, only the coded identity associated with documents or other participant data may be copied (obscuring any personally identifying information). Authorized representatives as noted above are bound to maintain the strict confidentiality of medical and research information that may be linked to identify individuals. The clinical site will normally be notified in advance of auditing visits.

15.4 Informed Consent

The consent process will be conducted by qualified study personnel (the Study Coordinator and/or Investigator or other designee). All participants (or their legally acceptable representative) must read, sign and date a consent form prior to participation in the study, and/or undergoing any study-specific procedures.

The informed consent form must be updated or revised whenever there is new clinically significant information applicable to the safety of the participants when a protocol amendment is

indicated, and/or whenever any new information becomes available that may affect an individual's participation in the study.

15.5 Study Subject Confidentiality

The study participant's contact information will be securely stored at each clinical site for internal use during the study. At the end of the study, all records will continue to be kept in a secure location for as long a period as dictated by local IRB and Institutional regulations.

Study participant research data, which is for purposes of statistical analysis and scientific reporting, will be transmitted to and stored at the GRADE Coordinating Center. This will not include the participant's contact or identifying information. Rather, individual participants and their research data will be identified by a unique study identification number that cannot be used to identify any individual subject. The study data entry and study management systems used by clinical sites and by Coordinating Center research staff will be secured and password protected. At the end of the study, all study databases will be de-identified and archived at the Coordinating Center.

A Certificate of Confidentiality will be obtained from the NIH.

15.6 Sample and Data Storage

Data collected for this study will be analyzed and stored at the GRADE Coordinating Center. After the study is completed, the de-identified, archived data will be transmitted to and stored at the NIDDK Data Repository, under the supervision of the NIDDK/NIH, for use by other researchers including those outside of the GRADE Study. Permission to transmit data to the NIDDK Data Repository will be included in the informed consent.

With the participant's approval and as approved by local IRBs, de-identified biological samples will be stored at the NIDDK Biosample Repository with the same goal as the sharing of data with the NIDDK Data Repository. These samples could be used for research into the causes of diabetes and obesity, its complications and other conditions for which individuals with diabetes are at increased risk and to improve treatment. The Repository will also be provided with a code-link that will allow linking the biological specimens with the phenotypic data from each participant, maintaining the masking of the identity of the participant.

During the conduct of GRADE, an individual participant can choose to withdraw consent to have biological specimens stored for future research. However, withdrawal of consent with regard to biosample storage will not be possible after GRADE is completed.

Once GRADE funding is completed, access to study data and/or samples will be provided through the NIDDK Repository.

15.7 Preservation of the Integrity of the Study

The scientific integrity of the trial dictates that results be reported on a study-wide basis; thus, an individual site will not report the data collected from its site alone. All presentations and publications using GRADE study data must protect the main objectives of the trial. Specifically, all presentations and publications will be generated and coordinated so that the main objectives of the trial are not compromised, such as might occur by early or ill-timed publication of an ancillary study. Data that could be perceived as threatening the equipoise of the trial will not be

disclosed prior to release of the primary study outcomes. Timing of presentations or publications of data and the venue where they will be presented or published will be determined or approved by the GRADE Publications and Presentations Committee. Study results should be discussed with the news media only upon authorization of the Publications and Presentations Committee and the Executive Committee, and never before the results are presented. Any written statements about this study that are shared with national media should be approved by the Publications and Presentations Committee and/or Executive Committee before release.

15.8 STUDY TIMELINE

The total duration of the study is projected to be approximately 10 years with the following approximate timeline. The first 6 months will be devoted to start-up. Recruitment will start in 2013 and will end approximately 3.5 years later in 2017 under the revised study plan implemented with Protocol v.1.6. All participants will be followed until the end of study, approximately 7.5 years after the randomization of the first participant. The earliest participants enrolled in the study will be followed for up to about 7.5 years and the last participant for up to approximately 4 years. The remaining 15 months of GRADE will be devoted to final database closure and archiving, and to statistical analysis and presentation of results.

16.0 STUDY ADMINISTRATION

16.1 STUDY LEADERSHIP AND GOVERNANCE

The **Steering Committee** will govern the conduct of the study. The Steering Committee will be composed of the Study Chairman, the PI of the Coordinating Center, representatives of the sponsoring NIH Institute (NIDDK), the principal investigators of the clinical sites, chairperson of the Study Coordinators subcommittee, and the PI of the Central Biochemistry Laboratory. The Steering Committee will meet in person at least annually.

An **Executive Committee** will be composed of the Study Chairman, Vice-Chairperson(s), PI of the Coordinating Center, the Project Director at the Coordinating Center, the Chair of the Study Coordinators Committee, and the NIDDK Project Scientist and Program Official. The Executive Committee will manage the day-to-day conduct of the study. It will meet by phone regularly, with administrative and other support staff in attendance to discuss the progress of the study and provide frequent guidance and supervision. Members of the Steering Committee and chairpersons of the Committees and subcommittees who are managing specific areas of the protocol will also join the Executive Committee call, as needed.

Conflict of Interest policy: The independence of this study from any actual or perceived influence by the pharmaceutical industry is critical and, therefore, the pharmaceutical industry did not and will not play any role in the design, conduct, analysis, publication, or any aspect of this trial. The study leadership in conjunction with the NIDDK has established a policy and procedures for all study group members to disclose all conflicts of interest and will establish a mechanism for the management of all reported dualities of interest.

16.2 SUBCOMMITTEES

In addition to the Steering and Executive Committees, several subcommittees will be established to help conduct the study according to the protocol. These Subcommittees will include, among others, the following: Study Coordinators, Recruitment/Retention, Outcomes,

Protocol Oversight, Publication and Presentations, and Ancillary Studies. Working groups will be constituted within these subcommittees to address specific study issues.

16.3 Coordinating Center and Chairman's Office

The Coordinating Center will be located at the Biostatistics Center of The George Washington University under the direction of Dr. John Lachin. The Coordinating Center will be responsible for study support and management, and for data storage, management, and statistical analysis.

The Chairman's Office will be located at the Massachusetts General Hospital under direction of the Study Chair, Dr. David M. Nathan. The Chairman will provide overall administrative, clinical and scientific leadership for the conduct of GRADE.

Drs. Nathan and Lachin are both designated as a Principal Investigator for the NIDDK grant that supports GRADE.

16.4 Data and Safety Monitoring Board

A Data and Safety Monitoring Board (DSMB) will be established by the sponsoring NIH Institute, NIDDK, to oversee the safety and other aspects of the study.

BIBLIOGRAPHY AND REFERENCES CITED

1. Centers for Disease Control and Prevention. National diabetes fact sheet, 2011. http://www.cdc.gov/diabetes/pubs/pdf/ndfs_2011.pdf. Updated 2011. Accessed February 8, 2013.
2. Nathan DM. Long-term complications of diabetes mellitus. *N Engl J Med* 1993; 328:1676-1685.
3. Kannel WB MD. Diabetes and cardiovascular disease: The Framingham study. *JAMA*. 1979;241(19):2035-2038.
4. American Diabetes Association. Economic costs of diabetes in the U.S. in 2007. *Diabetes Care*. 2008;31(3):596-615.
5. Alexander GC, Sehgal NL, Moloney RM, Stafford RS: National trends in treatment of type 2 diabetes mellitus, 1994-2007. *Arch Intern Med.* 2008; 168(19):2088-2094.
6. Knowler WC, Barrett-Connor E, Fowler SE, et al; for Diabetes Prevention Program Research Group. Reduction in the incidence of type 2 diabetes with lifestyle intervention or metformin. *N Engl J Med*. 2002;346(6):393-403.
7. Tuomilehto J, Lindstrom J, Eriksson JG, et al; for Finnish Diabetes Prevention Study Group. Prevention of type 2 diabetes mellitus by changes in lifestyle among subjects with impaired glucose tolerance. *N Engl J Med*. 2001;344:1343-1350.
8. Gerstein HC, Yusuf S, Bosch J, et al; for DREAM Trial Investigators. Effect of rosiglitazone on the frequency of diabetes in patients with impaired glucose tolerance or impaired fasting glucose: A randomised controlled trial**.** *Lancet*. 2006;368(9541):1096-1105
9. The Diabetes Control and Complications Trial Research Group. The effect of intensive treatment of diabetes on the development and progression of long-term complications in insulin-dependent diabetes mellitus. *N Engl J Med*. 1993;329:977-986.
10. UK Prospective Diabetes Study (UKPDS) Group: Effect of intensive blood-glucose control with metformin on complications in overweight patients with type 2 diabetes (UKPDS 34). *Lancet.* 1998; 352(9131):854-865.
11. Holman RR, Paul SK, Bethel MA, et al. 10-year follow-up of intensive glucose control in type 2 diabetes. *N Engl J Med.* 2008; 359:1577-1589.
12. Nathan DM: Finding new treatments for diabetes - how many, how fast ... how good? *N Engl J Med.* 2007; 356(7):437-440.
13. Nathan DM: The impact of clinical trials on the treatment of diabetes mellitus. *J Clin Endocrinol Metab.* 2002; 87(5):1929-1937.
14. Preis SR, Hwang SJ, Coady S, et al. Trends in all-cause and cardiovascular disease mortality among women and men with and without diabetes mellitus in the framingham heart study, 1950 to 2005. *Circulation*. 2009;119(13):1728-1735.
15. American Diabetes Association: Standards of medical care in diabetes - 2017. *Diabetes Care* 40 (Suppl 1).
16. Inzucchi SE, Bergenstal RM, Buse JB, et al. Management of hyperglycemia in type 2 diabetes: A patient-centered approach: position statement of the American Diabetes Association (ADA) and the European Association for the Study of Diabetes (EASD). *Diabetes Care.* 2012; 35(6):1364-1379.
17. Nathan DM, Buse JB, Davidson MB, et al. Medical management of hyperglycemia in type 2 diabetes mellitus: a consensus algorithm for the initiation and adjustment of therapy: a consensus statement from the American Diabetes Association and the European Association for the Study of Diabetes. *Diabetes Care.* 2006;29(8):1963-1972.
18. American Association of Clinical Endocrinologists. AACE medical gudelines for clinical practice for the management of diabetes mellitus. *Endocr Pract.* 2007;13(suppl 1);19-22.

19. Kahn SE, Haffner SM, Heise MA, et al. Glycemic durability of rosiglitazone, metformin, or glyburide monotherapy. *N Engl J Med.* 2006;355:2427-2443.
20. Ryan DH, Espeland MA, Foster, GD, et al; for Look AHEAD Research Group. Look AHEAD (Action for Health in Diabetes): design and methods for a clinical trial of weight loss for the prevention of cardiovascular disease in type 2 diabetes. *Control Clin Trials.* 2003;24(5):610-628.
21. Collins F. Oral presentation at the "Comparative effectiveness and personalized medicine: An essential interface" conference. October 19-21, 2010, National Institutes of Health, Bethesda, MD.
22. Smith RJ, Nathan DM, Arslanian SA, Groop L, Rizza RA, Rotter JI. Individualizing therapies in type 2 diabetes mellitus based on patient characteristics: what we know and what we need to know. *J Clin Endocrinol Metabol*. 2010;95(4):1566-1574.
23. Kooy A, de Jager J, Lehert P, et al. Long-term effects of metformin on metabolism and microvascular and macrovascular disease in patients with type 2 diabetes mellitus. *Arch Intern Med.* 2009;169(6):616-625.
24. Piccinni C, Motola D, Marchesini G, Poluzzi E. Assessing the association of pioglitazone use and bladder cancer through drug adverse event reporting. *Diabetes Care.* 2011; 34(6):1369-1371.
25. Gerstein HC, Miller ME, Byington RP, et al; for Action to Control Cardiovascular Risk in Diabetes Study Group. Effects of intensive glucose lowering in type 2 diabetes. *N Engl J Med*. 2008;358(24):2545-2559.
26. Bonds DE, Miller ME, Bergenstal RM, et al; for Action to Control Cardiovascular Risk in Diabetes Study Group.The association between symptomatic, severe hypoglycaemia and mortality in type 2 diabetes: retrospective epidemiological analysis of the ACCORD study. *BMJ*. 2010;340:b4909.
27. Riddle MC, Ambrosius WT, Brillon DJ, et al. Epidemiologic relationships between A1C and all-cause mortality during a median 3.4-year follow-up of glycemic treatment in the ACCORD trial. *Diabetes Care*. 2010;33(5):983-990.
28. Gangji AS, Cukierman T, Gerstein HC, Goldsmith CH, Clase CM. A systematic review and meta-analysis of hypoglycemia and cardiovascular events: a comparison of glyburide with other secretagogues and with insulin. *Diabetes Care.* 2007;30(2):389-394.
29. Goldstein BJ, Feinglos MN, Lunceford JK, Johnson J, Williams-Herman DE; for Sitagliptin 036 Study Group. Effect of initial combination therapy with sitagliptin, a dipeptidyl peptidase-4 inhibitor, and metformin on glycemic control in patients with type 2 diabetes. *Diabetes Care.* 2007;30(8):1979-1987.
30. Scheen AJ, Charpentier G, Ostgren CJ, Hellqvist A, Gause-Nilsson I. Efficacy and safety of saxagliptin in combination with metformin compared with sitagliptin in combination with metformin in adult patients with type 2 diabetes mellitus. *Diabetes Metab Res Rev*. 2010; 26(7):540-9.
31. Taskinen MR, Rosenstock J, Tamminen I, et al. Safety and efficacy of linagliptin as add-on therapy to metformin in patients with type 2 diabetes: a randomized, double-blind, placebo-controlled study. *Diabetes Obes Metab*. 2011;13(1):65-74.
32. DeFronzo RA, Ratner RE, Han J, Kim DD, Fineman MS, Baron AD. Effects of exenatide (exendin-4) on glycemic control and weight over 30 weeks in metformin-treated patients with type 2 diabetes. *Diabetes Care*. 2005;28(5):1092-1100.
33. Buse JB, Rosenstock J, Sestig G, et al; for LEAD-6 Study Group. Liraglutide once a day versus exenatide twice a day for type 2 diabetes: a 26-week randomised parallel-group, multinational, open-label trial (LEAD-6). *Lancet.* 2009;374(9683):39-47.
34. Diamant M, Van GL, Stranks S, et al. Once weekly exenatide compared with insulin glargine titrated to target in patients with type 2 diabetes (DURATION-3): an open-label randomised trial. *Lancet*. 2010;375(9733):2234-2243.

35. Gerstein HC, Yale JF, Harris SB, Issa M, Stewart JA, Dempsey E. A randomized trial of adding insulin glargine vs. avoidance of insulin in people with Type 2 diabetes on either no oral glucose-lowering agents or submaximal doses of metformin and/or sulphonylureas. The Canadian INSIGHT (Implementing New Strategies with Insulin Glargine for Hyperglycaemia Treatment) Study. *Diabet Med.* 2006;23(7):736-742.
36. Holman RR, Cull CA, Turner RC. A randomized double-blind trial of acarbose in type 2 diabetes shows improved glycemic control over 3 years (U.K. Prospective Diabetes Study 44). *Diabetes Care.* 1999;22(6):960-964.
37. Black C, Donnelly P, McIntyre L, Royle PL, Shepherd JP, Thomas S. Meglitinide analogues for type 2 diabetes mellitus. *Cochrane Database Syst Rev*. 2007;(2);CD004654.
38. Holman RR, Farmer AJ, Davies MJ, et al; for 4-T Study Group. Three-year efficacy of complex insulin regimens in type 2 diabetes. *N Engl J Med*. 2009;361(18):1736-1747.
39. Bays HE, Goldberg RB, Truitt KE, Jones MR. Colesevelam hydrochloride therapy in patients with type 2 diabetes mellitus treated with metformin: glucose and lipid effects. *Arch Intern Med.* 2008;168(18):1975-1983.
40. Pijl H, Ohashi S, Matsuda M, et al. Bromocriptine: a novel approach to the treatment of type 2 diabetes. *Diabetes Care.* 2000;23(8):1154-1161.
41. Bartnik M, Ryden L, Ferrari R, et al. The prevalence of abnormal glucose regulation in patients with coronary artery disease across Europe. *Eur Heart J.* 2004;25:1880-1890.
42. Grassi MA, Mazzulla DA, Knudtson MD, et al. Patient self-report of prior laser treatment reliably indicates presence of severe diabetic retinopathy. *Am J Ophthalmol*. 2009;147:501-504.
43. Kaplan RM, Ganiats TG, Sieber WJ, Anderson JP. The quality of well-being scale: critical similarities and and differences with SF-36. *Int J Qual Health Care*. 1998;10:509-520.
44. DPPOS Research Group. DPPOS Neurocognitive Function Battery. DPPOS website. https://www.bsc.gwu.edu/dpp/neuromoo.pdf. Published September 30, 2011. Accessed November 9, 2012.
45. Wallace TM, Levy JC, Matthews DR. Use and abuse of HOMA modeling. *Diabetes Care.* 2004;27:1487-1495.
46. Utzschneider KM, Prigeon RL, Faulenbach MV, et al. Oral disposition index predicts the development of future diabetes above and beyond fasting and 2-h glucose levels. *Diabetes Care.* 2009;32:335-341.
47. Cobelli C, Toffolo GM, Dalla Man C, et al. Assessment of ß-cell function in humans, simultaneously with insulin sensitivity and hepatic extraction, from intravenous and oral glucose tests. *Am J Physiol Endocrinol Metab*. 2007;293:E1-E15.
48. Mari A, Schmitz O, Gastaldelli A, Oestergaard T, Nyholm B, Ferrannini E. Meal and oral glucose tests for assessment of beta-cell function: modeling analysis in normal subjects. *Am J Physiol Endocrinol Metab*. 2002;283:E1159-E1166.
49. Simon J, Gray A, Duley L and the Magpie Trial Collaborative Group. Cost-effectiveness of prophylactic magnesium sulphate for 9996 women with pre-eclampsia from 33 countries: economic evaluation of the Magpie Trial. *BJOG*. 2006;113:144-151.
50. D'Agostino RB, Sr, Vasan RS, Pencina MJ, et al. General cardiovascular risk profile for use in primary care. *Circulation.* 2008;117:743-753.
51. Stevens RJ, Kothari V, Adler AI, Stratton IM and the United Kingdom Prospective Diabetes Study (UKPDS) Group. The UKPDS risk engine: a model for the risk of coronary heart disease in Type II diabetes. *Clin Sci*. 2001;101:671-679.
52. Tomlinson G, Detsky AS. Composite end points in randomized trials: there is no free lunch. *JAMA*. 2010;303:267-268.
53. Ferreira-Gonzalez I, Busse JW, Heels-Ansdell D, et al. Problems with use of composite end points in cardiovascular trials: systematic review of randomised controlled trials. *BMJ*. 2007;334:786.

54. Martin CL, Albers J, Herman WH and the DCCT/EDIC Research Group. Neuropathy among the diabetes control and complications trial cohort 8 years after trial completion. *Diabetes Care.* 2006;29:340-344.
55. Lachin, JM. *Biostatistical Methods: The Assessment of Relative Risks, Second Edition*. Hoboken, NJ: John Wiley & Sons, Inc., 2010.
56. Holm S. A simple sequentially rejective multiple test procedure. Scand J Statist 1989;6:65-70.
57. Liang KY, Zeger SL. Longitudinal data analysis using generalized linear models. *Biometrika.* 1986;73:13-22.
58. Demidenko, E. *Mixed Models: Theory and Applications*. Hoboken, NJ: John Wiley & Sons, Inc., 2004.
59. O'Brien PC. Procedures for comparing samples with multiple endpoints. *Biometrics*. 1984; 40:1079-1087.
60. Lachin JM. Applications of the Wei-Lachin multivariate one-sided test for multiple outcomes on possibly different scales (2014) *PLoS ONE*, 9 (10), art. no. e108784.
61. Lachin JM, Foulkes MA. Evaluation of sample size and power for analyses of survival with allowance for nonuniform patient entry, losses to follow-up, noncompliance, and stratification. *Biometrics*. 1986;42:507-519.
62. Lachin JM, Viberti G, Zinman B; for ADOPT Study Group. Renal function in type 2 diabetes with rosiglitazone, metformin, and glyburide monotherapy. *Clin J Am Soc Nephrol*. 2011;6:1032-1040.
