# Supplementary material for: Glycemia reduction in type 2 diabetes—Hypoglycemia outcomes: A randomized clinical trial
Source: PLoS One. 2024 Nov 15;19(11):e0309907. doi: 10.1371/journal.pone.0309907 (PMC11567630; doi:10.1371/journal.pone.0309907)
Supplement: S2 Text — (DOCX) [file pone.0309907.s003.docx]

# Text S2: GRADE Masking and Random Assignment

Masking: GRADE was an unmasked trial. The participants and clinic staff were aware of the treatment assignments; however, the investigators at the laboratories and reading centers and the members of the adjudication committee were unaware of the treatment assignments and the identity of each participant.

Random Assignment:

GRADE comprised 37 clinical centers and 8 affiliated sub-sites that enrolled and followed the cohort of 5047 participants. The random assignment for a participant at a sub-site was generated from the random sequence of assignments for the corresponding parent site. Random assignments were generated for 40 sites with three extra. The 38^th^ and 39^th^ sequences were later applied when two new clinics were added. Each clinic sequence consisted of 200 assignments to allow for some sites to exceed the average of 136 participants per center. Six sites (2, 3, 10, 13, 18, 24, 29) later exceeded the 200 maximum and for these the randomized assignments were extended to 300 allocations.

The randomization sequence for each site started with a random block of 12 assignments (3 blocks, each with one assignment to each of the 4 treatment arms). The 13^th^ and subsequent assignments were then generated by an Urn Design with parameter values alpha=3 and beta=10. See Reference [1]. The urn design random allocation was applied so long as treatment balance was maintained within acceptable limits. Let m denote the current number of participants enrolled. And of these let N_i(m) and N_j(m) denote the numbers assigned to date for a given pair of groups (ij =1,2,3,4). The (m+1)th-assignment is generated from the UD-so long as max |N_i(m) – N_j(m)| <= 3. If at any allocation step max |N_i(m) – N_j(m)| > 3, then a deterministic allocation is applied to restore treatment imbalance within acceptable limits. Such a maximum tolerated imbalance procedure was also employed to determine the next allocation so as to ensure that <= 3 allocations are made in a row to the same group, no more than 10 assignments are made without a single assignment to any group, and there is no greater than 10% imbalance at 136 assignments for any group within any site, tapering down as n increases. A custom in-house program was employed.

Rosenberger, W. and Lachin J.M. *Randomization in Clinical Trials: Theory and Practice*. Second Edition. John Wiley and Sons, 2016. (ISBN: 978-1-118-74224-2)
