# Supplementary material for: Glycemia reduction in type 2 diabetes—Hypoglycemia outcomes: A randomized clinical trial
Source: PLoS One. 2024 Nov 15;19(11):e0309907. doi: 10.1371/journal.pone.0309907 (PMC11567630; doi:10.1371/journal.pone.0309907)
Supplement: S3 Text — A. Hypoglycemia data collection. B. Severe hypoglycemia adjudication. C. Hypoglycemia definitions. (DOCX) [file pone.0309907.s004.docx]

# **Text S3. Hypoglycemia data collection and definitions**

1. **Hypoglycemia Data Collection:** At each quarterly visit, the following questions related to hypoglycemia were collected on the participant-completed SYMPTOM form, then data were entered by study staff.

**GRADE Symptom and Glucose Measurement Form**

| In the last 30 days, have you had any of the following? **(Check only one per question)** | | |
| --- | --- | --- |
| 1. Any episodes of low blood sugar symptoms (for example, otherwise unexplained sweating, heart racing, and/or confusion) that got better with glucose or food? | Yes  1 | No  2 |
| **If YES,** a. How many times did you have these low blood sugar episodes? | Time(s) | |
| b. Did you check your blood sugar level during these episodes? | Yes  1 | No  2 |
| **If YES,**  1) Was your blood sugar level less than 70 mg/dl on any occasion? | Yes  1 | No  2 |
| **If YES,** a) Was your blood sugar level less than 54 mg/dl on any occasion? | Yes  1 | No  2 |
| 1. Any blood sugar level by finger-stick less than 70 mg/dl, but without low blood sugar symptoms (for example, otherwise unexplained sweating, heart racing, and/or confusion)? | Yes  1 | No  2 |
| **If YES,**  a. Was your blood sugar level less than 54 mg/dl on any occasion? | Yes  1 | No  2 |
| 1. Severe low blood sugar episode during which you could not treat yourself and required help from someone else (family member, friend or medical personnel) to treat? | Yes  1 | No  2 |
| **If YES,** a. How many times did you have these severe low blood sugar episodes? | Time(s) | |
| b. Did any of these episodes result in seizure or loss of  consciousness? | Yes  1 | No  2 |
| c. Did any of these episodes result in injury to yourself or   others? | Yes  1 | No  2 |

1. **Severe hypoglycemia adjudication:** At each quarterly visit, the study staff collected data on severe hypoglycemia on the Main Visit Form. A “YES” response to severe hypoglycemia led to a request for more information (see below).

**GRADE Main Visit Form**

| **Since the last visit,** have you had: |  |  |
| --- | --- | --- |
| B.1.g. Severe hypoglycemia (another person helped them to treat the episode, usually because the person was confused or otherwise impaired and could not treat him/herself)? | Yes  1 | No  2 |

If severe hypoglycemia was reported at any time, study staff were asked to submit detailed information about the event, including pertinent medical records, to the Severe Hypoglycemia Adjudication Subcommittee. Two adjudicators from the subcommittee were assigned by the Coordinating Center to determine if the event was severe hypoglycemia based on the criteria for severe hypoglycemia (see below), while considering any other contextual information provided by the medical records. In the case of a disagreement, a third adjudicator would break the tie.

**GRADE Adjudication Criteria for Severe Hypoglycemia**

| Criteria |
| --- |
| 1. Event required assistance from a third party to resolve |
| 1. Event required treatment with glucagon or IV glucose |
| 1. Event resulted in injury to the participant or others (e.g., a motor vehicle accident in which the participant was the driver) |
| 1. Event included loss of consciousness or seizure |

1. **Hypoglycemia Categories**

| **Hypoglycemia** | **Data Collection** | **Description** |
| --- | --- | --- |
| Severe hypoglycemia | Positively adjudicated hypoglycemia | Event required assistance, glucagon or IV glucose, resulted in injury, or included loss of consciousness, per participant report, and the adjudication committee **did** provide a positive adjudication, regardless of what was reported on the SYMPTOM form. *(Event occurring at any time)* |
| Hypoglycemic symptoms | YES to SYMPTOM Q7, Q9 **OR** positively adjudicated hypoglycemia | Event with low blood sugar symptoms, which got better with food  *(only collected in past 30 days).*  **OR**  Event that required assistance, glucagon or IV glucose, resulted in injury, or included loss of consciousness, per participant report. *(Event occurring at any time)* |
| BG between 70-54^a^ | YES to SYMPTOM Q7.A.B.1 **AND**  NO to SYMPTOM Q7.A.B.2  **OR**  YES to SYMPTOM Q8 AND  NO to SYMPTOM Q.8.A | Participant reported measuring their blood glucose <70, but greater than or equal to 54, with or without symptoms of hypoglycemia  *(only collected in past 30 days).* |
| BG less than 54 ^a^ | YES to SYMPTOM Q7.A.B.2  **OR**  YES to SYMPTOM Q.8.A | Participant reported measuring their blood glucose <54, with or without symptoms of hypoglycemia  *(only collected in past 30 days).* |
| Symptoms only | YES to SYMPTOM Q7 **AND**  [NO to SYMPTOM Q7.B **OR** Q7.B.1] **AND**  NO to SYMPTOM Q8 **AND**  NO to SYMPTOM Q9 **AND**  no positively adjudicated hypoglycemia | Participant reported symptoms of hypoglycemia  *(only collected in past 30 days)*  but did not have a reported blood glucose <70,  *(only collected in past 30 days)*  and did not meet the criteria for “any severe” hypoglycemia (described in line 1, above). |
| Any hypoglycemia | YES to SYMPTOM Q7, Q8, Q9 **OR** positively adjudicated hypoglycemia | Event with low blood sugar symptoms, which got better with food  *(only collected in past 30 days).*  **OR**  Measured blood glucose <70  *(only collected in past 30 days).*  **OR**  Event that required assistance, glucagon or IV glucose, resulted in injury, or included loss of consciousness. *(Event occurring at any time)* |
| No Hypoglycemia | NO to SYMPTOM Q7, Q8, Q9 **AND** no positively adjudicated hypoglycemia | Participant did not meet the definition of “any hypoglycemia” (described above). |

^a^Within the manuscript text, “measured hypoglycemia” refers to any hypoglycemia where the participant reported their blood glucose measured <
