# Supplementary material for: Glycemia reduction in type 2 diabetes—Hypoglycemia outcomes: A randomized clinical trial
Source: PLoS One. 2024 Nov 15;19(11):e0309907. doi: 10.1371/journal.pone.0309907 (PMC11567630; doi:10.1371/journal.pone.0309907)
Supplement: S1 Table — (DOCX) [file pone.0309907.s005.docx]

| **Institution** | **City, State** | **Name of IRB** | **IRB Project Number** |
| --- | --- | --- | --- |
| Albert Einstein College of Medicine | Bronx, NY | Albert Einstein College of Medicine IRB | 2012-601 |
| Atlanta VA Medical Center | Decatur, GA | Emory University IRB | IRB00064284 |
| Baylor College of Medicine | Houston, TX | IRB for Baylor College of Medicine and Affiliated Hospitals | H-32387 |
| Baylor Scott & White Research Institute (Baylor Research Institute) | Dallas, TX | Baylor Scott & White Research IRB | 013-025 |
| Case Western Reserve University/Cleveland VA/MetroHealth Medical Center | Cleveland, OH | VA Northeast Ohio Healthcare System (VANEOHS) IRB | 1583928 |
|  |  | The MetroHealth System IRB | IRB00000685 |
| Columbia University Medical Center | New York, NY | Columbia University IRB | IRB-AAAL0052 |
| Duke University Medical Center | Durham, NC | Duke University Health System IRB | Pro00044255 |
| Indiana University | Indianapolis, IN | Indiana University IRB | 1302010611 |
| International Diabetes Center | Minneapolis, MN | HealthPartners Institute IRB | 4282-13-A |
| Kaiser Permanente Northwest | Portland, OR | Kaiser Permanente Interregional IRB | 1393280 |
| Kaiser Permanente of Georgia | Duluth, GA  Atlanta, GA | Kaiser Permanente Georgia IRB | 1306942 |
| Massachusetts General Hospital | Boston, MA | Mass General Brigham IRB | 2012P002605 |
| MedStar Health Research Institute/ MedStar Baltimore | Hyattsville, MD  Baltimore, MD | MedStar Health Research Institute IRB | 2013-033 |
| Miami VA Healthcare System/University of Miami | Miami, FL | Miami VA Healthcare System Human Studies Subcommittee | 1161643 |
|  |  | University of Miami Human Subjects Research Office | 20130049 |
| Oregon Health & Science University | Portland, OR | OHSU IRB | MIRB#3697 |
| Pacific Health Research and Education Institute/VA Pacific Islands | Honolulu, HI | VA Pacific Islands Health Care Systems IRB | 2013-02 |
| Pennington Biomedical Research Center | Baton Rouge, LA | Pennington Biomedical Research Center IRB | 13003-PBRC |
| San Diego VA Medical Center | San Diego, CA | VA San Diego Healthcare System IRB | H120103 |
| Southwestern American Indian Center | Phoenix, AZ | NIDDK/NIAMS IRB | 13-DK-N087 |
| St. Luke's‐Roosevelt Hospital | New York, NY | Icahn School of Medicine at Mount Sinai IRB | SLR 12-161 |
| SUNY Downstate Medical Center/NewYork-Presbyterian/Queens | Brooklyn, NY  Flushing, NY | SUNY DownstateIRB | 436303 |
|  |  | NewYork-Presbyterian/Queens IRB | 14400422 |
| The University of North Carolina Diabetes Care Center | Durham, NC | University of North Carolina at Chapel Hill, Office of Human Research Ethics, Biomedical IRB | 13-1108 |
| University of Alabama Birmingham | Birmingham, AL | University of Alabama at Birmingham IRB | F121219008 |
| University of Cincinnati/Cincinnati VA Medical Center | Cincinnati, OH | University of Cincinnati IRB | 2012-4712 |
| University of Colorado‐Denver/VA | Denver, CO | Colorado Multiple Institutional Review Board | 13-0131 |
| University of Iowa | Iowa City, IA | The University of Iowa IRB | 201607712  201301769 |
| University of Michigan | Ann Arbor, MI | University of Michigan Medical School IRB | HUM00066643 |
| University of Minnesota | Minneapolis, MN | University of Minnesota IRB | 1303M29544 |
| University of Nebraska Medical Center/Omaha VA | Omaha, NE | University of Nebraska Medical Center IRB | 049-13-FB |
|  |  | Omaha VA Medical Center IRB | 00859 |
| University of New Mexico | Albuquerque, NM | UNM Health Sciences Center Human Research Review Committee | 13-073 |
| UT Health San Antonio | San Antonio, TX | UT Health Science Center at San Antonio IRB | HSC20130205H |
| University of Texas‐Southwestern Medical Center | Dallas, TX | University of Texas Southwestern Medical Center IRB | STU 122012-025 |
| VA Puget Sound Health Care System/University of Washington | Seattle, WA | VA Puget Sound IRB | 1587515 |
| Vanderbilt University | Nashville, TN | Vanderbilt University IRB | 150534 |
| Washington University | St. Louis, MO | Washington University in St. Louis IRB | 201305080 |
| Yale University/Fair Haven Community Health Center/West Haven VA Medical Center | New Haven, CT | Yale University IRB | 1301011390 |
|  |  | VA Connecticut Healthcare System Human Studies Subcommittee | 1583087 |
